# Cumulative Metformin Use and Hepatocellular Carcinoma Risk After HCV SVR: A Multicentre Cohort Study

**DOI:** 10.1111/liv.70798

**Published:** 2026-07-24

**Authors:** Henar Calvo‐Sánchez, Lorena Jara‐Fernández, Raquel Encijo‐Heredia, Irene Villarino, Rubén Alvarado, Marta Quiñones‐Calvo, María‐Luisa Gutiérrez, Joaquín Miquel, Miguel Torralba, Myriam Catalá, José Gómez, Sonia Albertos, Óscar Barquero‐Pérez, Conrado Fernández‐Rodríguez, Juan‐Ramón Larrubia

**Affiliations:** ^1^ Service of Gastroenterology University Hospital of Guadalajara Guadalajara Spain; ^2^ Translational Research Group on Cellular Immunology (GITIC) Instituto de Investigación Sanitaria de Castilla‐La Mancha (IDISCAM) Guadalajara Spain; ^3^ Department of Medicine and Medical Specialties University of Alcalá, Alcalá de Henares Madrid Spain; ^4^ Service of Gastroenterology University Hospital Fundación Alcorcón Madrid Spain; ^5^ Service of Internal Medicine University Hospital of Guadalajara Guadalajara Spain; ^6^ Department of Biology and Geology, Physics and Inorganic Chemistry, ESCET Rey Juan Carlos University Madrid Spain; ^7^ Institute for Global Change Research (IICG‐URJC), Rey Juan Carlos University Madrid Spain; ^8^ Gastroenterology Service Alt Penedès–Garraf Sanitary Complex Barcelona Spain; ^9^ Department of Signal Theory and Communications and Telematic Systems and Computing Rey Juan Carlos University Madrid Spain; ^10^ Department of Public Health and Medical Specialties Rey Juan Carlos University Madrid Spain

**Keywords:** causal inference, hepatitis C treatment, inverse probability weighting, portal hypertension, risk stratification, type 2 diabetes mellitus

## Abstract

**Background:**

Although sustained virological response (SVR) after hepatitis C virus treatment reduces hepatocellular carcinoma (HCC) incidence, residual risk persists. Metformin has been associated with lower HCC risk, but whether cumulative metformin exposure (CME) lowers post‐SVR risk remains unclear. We evaluated whether CME was associated with lower HCC risk after SVR.

**Methods:**

We analysed a multicentre cohort of 1531 patients who achieved SVR after direct‐acting antiviral therapy (median follow‐up, 75.5 months). Metformin exposure was modelled as a time‐updated cumulative variable in start–stop Cox models to control for immortal‐time bias. Stabilised inverse probability weighting addressed confounding by indication and censoring. The overall model was sex‐stratified and adjusted for FIB‐4, clinically significant portal hypertension (CSPH), type 2 diabetes mellitus (T2DM) and smoking. A prespecified T2DM‐restricted analysis used T2DM‐specific IPTW plus IPCW, sex stratification and CSPH adjustment.

**Results:**

During follow‐up, 50 patients developed HCC. Crude incidence was highest among patients with FIB‐4 > 3.25, CSPH and T2DM without metformin exposure (4.84 cases per 100 person‐years). In the overall weighted model, CME was associated with lower HCC risk (HR, 0.46 per year; 95% CI, 0.27–0.77; *p* = 0.004). CSPH, T2DM, smoking and FIB‐4 > 3.25 independently increased risk. In the T2DM‐restricted model, each additional year remained associated with lower HCC hazard (HR, 0.49; 95% CI, 0.29–0.84; *p* = 0.009), whereas CSPH was associated with higher risk (HR, 6.04; 95% CI, 2.12–17.19; *p* < 0.001).

**Conclusion:**

After SVR, CME was associated with lower HCC risk; this possible duration‐dependent inverse association was clinically interpretable only among metformin‐eligible patients with T2DM.

## Key Points

- After sustained virological response (SVR), hepatocellular carcinoma (HCC) risk remains substantial with advanced fibrosis, clinically significant portal hypertension (CSPH), active smoking and type 2 diabetes mellitus (T2DM).
- Time‐updated cumulative metformin exposure was associated with lower post‐SVR HCC risk, with causal interpretation restricted to metformin‐eligible patients with T2DM.
- In the T2DM‐restricted weighted model, this inverse association persisted after adjustment for CSPH.
- Weighted time‐to‐event analyses support further evaluation of metformin as a potential chemopreventive adjunct after SVR.

## Lay Summary

People cured of hepatitis C can still develop liver cancer, especially with diabetes and advanced liver disease. Longer metformin use after cure was associated with lower liver cancer risk among people with diabetes. Metformin deserves further prevention studies, although regular surveillance remains essential.

**Abbreviations**
Akt: protein kinase B
ALBI: albumin‐bilirubin (grade/score)
ALT: alanine aminotransferase
AMPK: AMP‐activated protein kinase
APRI: AST to platelet ratio index
AST: aspartate aminotransferase
BMI: body mass index
CHC: chronic hepatitis C
CI: confidence interval
CME: cumulative metformin exposure
CSPH: clinically significant portal hypertension
DAA: direct‐acting antiviral
FIB‐4: fibrosis‐4 index
GGT: gamma glutamyl transpeptidase
HCC: hepatocellular carcinoma
HR: hazard ratio
INR: international normalised ratio
IPCW: inverse probability of censoring weighting
IPTW: inverse probability of treatment weighting
LSM: liver stiffness measurement
MELD‐Na: model for end‐stage liver disease–sodium
mTORC1: mechanistic target of rapamycin complex 1
NSAID: non‐steroid anti‐inflammatory drug
NSBB: non‐selective beta‐blocker
OLT: orthotopic liver transplantation
PH: proportional hazards
PI3K: phosphoinositide 3‐kinase
PS: propensity score
RSF: random survival forest
SE: standard error
SHAP: shapley additive explanations
SVR: sustained virological response
T2DM: type 2 diabetes mellitus

## 1 Introduction

Treatment of chronic hepatitis C (CHC) with direct‐acting antivirals (DAAs) has markedly reduced the incidence of hepatocellular carcinoma (HCC). Nevertheless, clinically relevant residual risk persists after sustained virological response (SVR), particularly among individuals with advanced fibrosis or clinically significant portal hypertension (CSPH) [1]. Although baseline fibrosis remains the cornerstone of HCC surveillance decisions [2], the availability of liver biopsy or elastography is often limited in real‐world practice. In such cases, non‐invasive serological scores such as FIB‐4 have proven useful in stratifying risk and guiding surveillance strategies [3, 4].

HCC risk within advanced fibrosis categories is heterogeneous, highlighting the need for additional modifiers of HCC risk. Beyond fibrosis alone, several host‐related factors could modulate HCC risk after SVR. Type 2 diabetes mellitus (T2DM) is a well‐established risk‐factor [5]. Given its central role in T2DM management, increasing interest has focused on the potential risk‐modifying effects of metformin [6, 7]. Experimental data support a plausible biological role of metformin, as it activates AMP‐activated protein kinase (AMPK), inhibits mechanistic target of rapamycin complex 1 (mTORC1) and promotes metabolic pathways that may counter hepatocarcinogenesis [8, 9, 10]. Nevertheless, clinical evidence remains inconsistent [11, 12, 13], partly because of the methodological limitations of previous observational studies.

A key challenge in assessing metformin effects is its time‐varying use, as fixed baseline exposure definitions are prone to immortal time bias. Consistent inference therefore requires dynamically updated exposure and appropriate adjustment for treatment assignment and censoring. However, prior studies have generally modelled metformin exposure as a fixed binary variable or broad exposure categories [6, 7], approaches that do not fully capture cumulative duration‐dependent effects. Moreover, evidence in post‐SVR European populations remains limited [6, 7].

We therefore analysed a multicentre cohort of patients with CHC who achieved SVR to determine whether cumulative metformin exposure (CME), modelled as a time‐updated variable within a weighted Cox proportional hazards framework, was associated with reduced HCC risk relative to established predictors, including advanced fibrosis, CSPH, T2DM and active smoking. As all metformin‐exposed patients had T2DM, causal interpretation was restricted to this population and based on a dedicated T2DM‐restricted weighted Cox model.

## 2 Patients and Methods

### 2.1 Patients and Study Design

**FIGURE 1:**
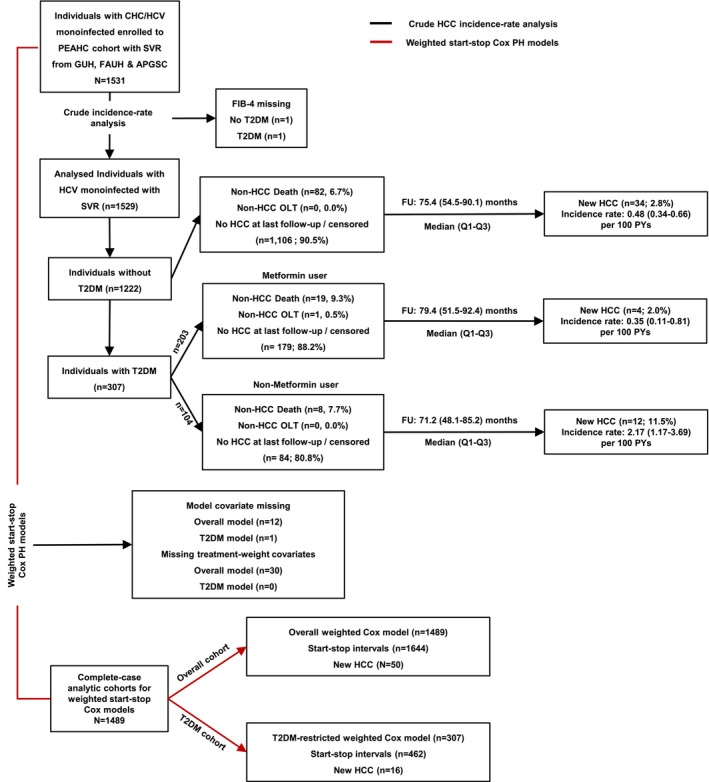
Study flowchart and analytic cohorts. Flowchart of patient selection from the PEAHC cohort. The study population corresponds to a predefined subcohort of 1531 individuals with chronic hepatitis C who achieved sustained virological response after direct‐acting antiviral therapy and were recruited from Guadalajara University Hospital (GUH), Fundación Alcorcón University Hospital (FAUH) and the Alto Penedès–Garraf Sanitary Complex (APGSC). The flowchart shows the derivation of the descriptive/incidence‐rate cohort and the complete‐case cohorts used for the weighted start–stop Cox proportional hazards analyses. Crude incidence‐rate analyses stratified by baseline FIB‐4 were conducted among 1529 patients with evaluable FIB‐4 and follow‐up data. Patients were first stratified according to the presence or absence of T2DM. Individuals with T2DM were further stratified according to metformin exposure during follow‐up. For each subgroup, follow‐up duration is reported as median (Q1–Q3). Outcome status is shown as incident hepatocellular carcinoma (HCC), non‐HCC death or non‐HCC orthotopic liver transplantation before HCC occurrence or no HCC at last follow‐up/censored. Complete‐case analytic cohorts for the weighted Cox models were derived according to the availability of variables required for propensity‐score estimation, IPCW estimation and outcome‐model fitting. The overall weighted Cox model included 1489 patients, represented by 1644 start–stop intervals, with 50 HCC events. The T2DM‐restricted weighted Cox model included 307 patients, represented by 462 start–stop intervals, with 16 HCC events. APGSC, Alto Penedès–Garraf Sanitary Complex; CHC, chronic hepatitis C; FAUH, Fundación Alcorcón University Hospital; GUH, Guadalajara University Hospital; HCC, hepatocellular carcinoma; HCV, hepatitis C virus; OLT, orthotopic liver transplantation; PY, person‐year; SVR, sustained virological response; T2DM, type 2 diabetes mellitus.

We conducted a retrospective analysis of prospectively collected data from 1531 patients with CHC who achieved SVR after DAA therapy at three Spanish centres: Guadalajara University Hospital, Fundación Alcorcón University Hospital and Alt Penedès‐Garraf Sanitary Complex. Data were recorded within the framework of the Spanish Strategic Plan for the Management of Hepatitis C (PEAHC) [14]. The study flowchart is shown in Figure 1. Sample size considerations are described in Supporting Information. For weighted analyses, complete‐case analytic cohorts were defined according to the availability of variables required for propensity‐score (PS) estimation, censoring‐weight estimation and outcome‐model fitting, yielding 1489 patients in the overall cohort and 307 patients in the T2DM‐restricted cohort.

Detailed inclusion and exclusion criteria, participating period, follow‐up procedures and HCC surveillance practices are provided in the Supporting Information. The primary outcome was incident HCC after SVR. The overall cohort was used to describe HCC incidence and to evaluate the main clinical determinants of post‐SVR HCC risk. Because metformin was clinically indicated only in patients with T2DM, the prespecified T2DM‐restricted weighted Cox model was considered the primary analysis for causal interpretation of the metformin association. The overall weighted Cox model was retained as a complementary etiologic and risk‐stratification analysis in the overall SVR cohort.

### 2.2 Baseline Variables

Baseline variables included: age, sex, body mass index (BMI), alcohol consumption (> 20 g/day), smoking status, ALT, AST, GGT, albumin, sodium, INR, bilirubin, creatinine, platelet count, T2DM status, metformin exposure, use of non‐selective beta‐blockers (NSBBs) or statins, liver stiffness (FibroScan), FIB‐4 [15], APRI, ALBI, MELD‐Na, cirrhosis and CSPH. Additional diabetes‐specific variables recorded in patients with T2DM included HbA1c at T2DM diagnosis, use of non‐metformin glucose‐lowering medications and estimated duration of diabetes.

For the primary analyses, CSPH was operationally defined using clinically available surrogate markers of portal hypertension derived from routine‐care data, including gastroesophageal varices, splenomegaly, ascites, encephalopathy, portal vein diameter > 12 mm or platelet count < 100 000/μL after excluding hematologic causes, consistent with clinically accepted surrogate markers of portal hypertension; its concordance with the Baveno VII liver stiffness‐based rule‐in criterion is reported in Supporting Information and Table S1. Cirrhosis was defined based on histology, transient elastography > 12.5 kPa or compatible clinical/imaging findings. T2DM was defined by a documented diagnosis in the medical record, use of glucose‐lowering therapy or diagnostic glycemic criteria, including fasting plasma glucose ≥ 126 mg/dL or 2‐h plasma glucose ≥ 200 mg/dL during an oral glucose tolerance test or HbA1c ≥ 6.5%. For metformin exposure, the start and end dates were extracted from clinical records, allowing the computation of cumulative exposure across the entire follow‐up period.

### 2.3 Estimation of Inverse Probability Weights

To address confounding by indication and potentially informative censoring, stabilised inverse probability of treatment (IPTW) and censoring (IPCW) weights were incorporated into the Cox proportional hazards (PH) analyses, consistent with inverse‐probability weighting methodology for confounding and selection‐bias adjustment [16]. Details on the strategy of estimation of inverse probability weights are shown in Supporting Information and Figures S1–S3.

### 2.4 Modelling of Metformin Exposure (Time‐Dependent Covariate)

Metformin use changes dynamically over time; therefore, exposure was modelled as a time‐updated covariate within a counting‐process (start–stop) Cox structure, allowing correct classification of unexposed and exposed person‐time and avoiding immortal‐time bias.

### 2.5 Multivariable Cox Proportional Hazards Models

A weighted start‐stop overall Cox PH model stratified by sex was fitted. Covariates were pre‐specified to provide a parsimonious clinically relevant adjustment set, given the limited number of HCC events. In addition to CME, the model included CSPH and FIB‐4 as markers of baseline liver disease severity, T2DM as both the clinical indication for metformin and a determinant of HCC risk and active smoking as an established independent HCC risk factor. Sex was incorporated as a stratification variable because preliminary evaluations indicated non‐proportionality. We used cause‐specific Cox models to estimate etiologic associations between covariates and HCC risk.

Sensitivity analyses were performed in the subgroup with baseline liver stiffness measurement (LSM) available. First, FIB‐4 was replaced by transient elastography‐derived LSM, modelled continuously per 5‐kPa increase. Second, the CSPH variable was replaced by fulfilment of the Baveno VII liver stiffness‐based rule‐in criterion for CSPH, defined as LSM ≥ 25 kPa [17].

To enhance the causal interpretability of the association between CME and HCC risk, we performed a prespecified analysis restricted to patients with T2DM, the population corresponding to the clinical indication for metformin use. In this T2DM‐only cohort, the previously described T2DM‐specific stabilised IPTW were combined with stabilised IPCW to account for treatment selection and potentially informative censoring. We then fitted a weighted start–stop Cox PH model with CME treated as a time‐dependent variable, stratified by sex and adjusted for CSPH.

To assess whether the association between CME and HCC risk was driven by extreme analytic weights, we repeated the final weighted Cox models after truncating total analytic weights at the 1st–99th and 5th–95th percentiles.

Additional details on models' specification and performance are provided in the Supporting Information and Table S2.

### 2.6 Fine‐Gray Sensitivity Analysis

As a sensitivity analysis addressing competing risks, Fine–Gray subdistribution hazard models were fitted for HCC in both the overall cohort and the prespecified T2DM‐restricted cohort, treating non‐HCC death and non‐HCC orthotopic liver transplantation (OLT) before HCC occurrence as competing events. Because CME was modelled in the primary analysis as an internal time‐updated exposure, its inclusion in a standard Fine–Gray subdistribution hazards model would not provide a straightforward and valid interpretation. Therefore, for this competing‐risk sensitivity analysis, metformin exposure was defined as post‐SVR ever metformin use. Adjusted cumulative incidence curves were obtained from the Fine–Gray model using model‐based predictions under prespecified covariate profiles.

### 2.7 Machine Learning–Based Exploratory Analyses

As a complementary, exploratory approach, Random Survival Forest (RSF) [18] with SHapley Additive exPlanations (SHAP) [19] were used to explore nonlinear associations and interactions. Details are provided in the Supporting Information.

### 2.8 Artificial Intelligence

A large language model (ChatGPT, OpenAI) was used exclusively to improve the readability and language (e.g., grammar and style) of the manuscript, under full human supervision. No artificial intelligence tool was used to generate scientific content, interpret the results or draw conclusions. Machine‐learning methods (RSF with SHAP) were used solely for exploratory analyses, as described in the Supporting Information.

### 2.9 Ethics Approval

Ethical approval for this study was obtained from the Research Ethics Committee of Guadalajara University Hospital (ACTA No. 1/2023) on January 17, 2023 and was subsequently endorsed by all participating centres. All the research was conducted in accordance with the Declarations of Helsinki. Owing to the retrospective study design and use of fully anonymised data, the requirement for written informed consent was waived by the ethics committees of all participating institutions.

## 3 Results

### 3.1 Clinical and Demographic Features According to T2DM and Metformin Use

The study cohort comprised 1531 patients with CHC who achieved SVR after DAA treatment. The median age was 57 years, 57% of patients were male, 26% had cirrhosis and 17% had CSPH. Overall, 20% of patients had T2DM, of whom approximately two thirds contributed at least one metformin‐exposed risk interval during follow‐up. The distribution of antidiabetic treatments among patients with T2DM according to metformin exposure during follow‐up is shown in Table S3.

Compared with non‐diabetic patients, individuals with T2DM—irrespective of metformin exposure—were older, had a slightly higher BMI and exhibited more advanced liver disease. This was reflected by a higher prevalence of cirrhosis and CSPH, higher FIB‐4 values and LSMs and lower platelet counts and serum albumin levels, although most laboratory parameters remained within the normal range.

Baseline characteristics were broadly comparable between the two T2DM subgroups, with and without metformin exposure. Patients not receiving metformin had a lower prevalence of statin use, lower serum albumin levels and higher GGT values than metformin‐treated patients. Despite this overall similarity, the crude proportion of HCC events was markedly higher among T2DM patients not treated with metformin, whereas the proportion among metformin‐treated patients was comparable to that observed in non‐diabetic individuals. Only one patient in the overall cohort underwent OLT for a non‐HCC indication before HCC occurrence. Median follow‐up duration and non–HCC‐related mortality were similar across the three groups (Table 1).

**TABLE 1:** Baseline demographic and clinical features and crude follow‐up outcomes according to type 2 diabetes mellitus status and metformin exposure during follow‐up.

	Total (*N* = 1531)	Non‐T2DM (*N* = 1223) [A]	T2DM (*N* = 308)		Global *p*	A vs. B *p*	A vs. C *p*	B vs. C *p*
			No‐met (*N* = 104) [B]	Met (*N* = 204) [C]				
Age (years)	57 (50–69)	56 (49–68)	60 (53–73)	64 (54–76)	**< 0.001** ^d^	**0.005** ^e^	**< 0.001** ^e^	NS^e^
Female sex (*N*, %)	657 (43)	542 (44)	38 (36)	77 (38)	NS^c^	—	—	—
BMI (kg/m^2^)	25.7 (23.2–28.7)	25.4 (22.9–28.1)	26.5 (24.3–30.0)	27.0 (24.5–31.2)	**< 0.001** ^d^	**0.009** ^e^	**< 0.001** ^e^	NS^e^
T2DM (*N*, %)	308 (20)	0 (0)	104 (100)	204 (100)	—	—	—	—
HbA1c (*N*, %)^b^								
≤ 7%	—	—	60 (57.7)	95 (46.6)	**—**	**—**	**—**	NS^c^
> 7% and ≤ 8%	—	—	20 (19.2)	47 (23.0)				
> 8%	—	—	24 (23.1)	62 (30.4)				
T2DM duration (years)	—	—	6.5 (3–11)	7.8 (4–12)	—	—	—	NS^e^
Alco > 20 g/day (*N*, %)	327 (21.8)	259 (21.7)	21 (20.4)	47 (23.0)	NS^c^	—	—	—
Smoking (*N*, %)	795 (52.3)	646 (53.2)	53 (52.0)	96 (47.1)	NS^c^	—	—	—
Metformin (*N*, %)^a^	204 (13.3)	0 (0)	0 (0)	204 (100)	—	—	—	—
Statins (*N*, %)	268 (17.5)	145 (11.9)	30 (28.8)	93 (45.6)	**< 0.001** ^c^	**< 0.001** ^c^	**< 0.001** ^c^	**0.005** ^c^
NSBB (*N*, %)	95 (6.3)	64 (5.3)	10 (10.0)	21 (10.3)	**0.006** ^c^	0.048^c^	**0.005** ^c^	NS^c^
Liver stiffness (kPa)	7.9 (5.8–12.1)	7.7 (5.6–11.8)	8.4 (6.4–15.4)	10.1 (6.6–17.3)	**< 0.001** ^d^	0.035^e^	**< 0.001** ^e^	NS^e^
Cirrhosis (*N*, %)	391 (25.5)	268 (21.9)	43 (41.3)	80 (39.2)	**< 0.001** ^c^	**< 0.001** ^c^	**< 0.001** ^c^	NS^c^
CSPH (*N*, %)	257 (16.8)	176 (14.4)	25 (24.0)	56 (27.6)	**< 0.001** ^c^	**0.009** ^c^	**< 0.001** ^c^	NS^c^
ALT (U/L)	54 (36–89)	53 (36–89)	56 (38–94)	54 (33–93)	NS^d^	—	—	—
AST (U/L)	44 (30–72)	43 (30–72)	48 (35–83)	43 (31–72)	NS^d^	—	—	—
GGT (U/L)	56 (30–108)	53 (29–103)	82 (46–164)	68 (37–120)	**< 0.001** ^d^	**< 0.001** ^e^	**0.003** ^e^	0.034^e^
Bilirubin (mg/dL)	0.68 (0.50–0.90)	0.65 (0.50–0.90)	0.70 (0.50–0.93)	0.69 (0.50–0.93)	NS^d^	—	—	—
Albumin (g/dL)	4.3 (4.1–4.6)	4.3 (4.1–4.6)	4.1 (3.7–4.4)	4.3 (4.0–4.6)	**< 0.001** ^d^	**< 0.001** ^e^	0.031^e^	**0.001** ^e^
Platelet count (×10^9^/L)	188 (142–234)	193 (146–236)	158 (98–215)	174 (125–221)	**< 0.001** ^d^	**< 0.001** ^e^	**0.001** ^e^	NS^e^
Creatinine (mg/dL)	0.8 (0.7–0.9)	0.8 (0.7–0.9)	0.8 (0.7–0.9)	0.8 (0.7–0.9)	NS^d^	—	—	—
Sodium (mEq/L)	141 (139–142)	141 (140–142)	140 (138–142)	140 (139–142)	**< 0.001** ^d^	**0.001** ^e^	**< 0.001** ^e^	NS^e^
INR (Units)	1.00 (0.96–1.07)	1.00 (0.96–1.06)	1.02 (0.96–1.09)	1.01 (0.96–1.09)	NS^d^	—	—	—
FIB‐4 (Units)	1.9 (1.2–3.3)	1.77 (1.15–2.99)	2.6 (1.5–5.9)	2.3 (1.4–3.9)	**< 0.001** ^d^	**< 0.001** ^e^	**< 0.001** ^e^	NS^e^
FIB‐4 (*N*, %)								
< 1.45	543 (35.5)	464 (38.0)	25 (24.0)	54 (26.5)	**< 0.001** ^c^	**< 0.001** ^c^	**0.002** ^c^	NS^c^
1.45–3.25	599 (39.2)	482 (39.4)	33 (31.7)	84 (41.7)				
> 3.25	387 (25.3)	276 (22.6)	46 (44.2)	65 (31.9)				
MELD‐Na score	6.7 (6.4–7.7)	6.7 (6.4–7.6)	7.1 (6.4–8.6)	6.9 (6.4–8.1)	**0.002** ^d^	**0.018** ^e^	**0.003** ^e^	NS^e^
MELD ≥ 14 (*N*, %)	29 (2.1)	18 (1.6)	4 (4.3)	7 (3.7)	NS^c^	—	—	—
APRI score	0.68 (0.41–1.35)	0.65 (0.40–1.27)	0.98 (0.45–2.29)	0.74 (0.44–1.62)	**0.002** ^d^	**0.001** ^e^	NS^e^	NS^e^
ALBI score	0.32 (0.19–0.42)	0.31 (0.18–0.42)	0.36 (0.24–0.44)	0.34 (0.23–0.43)	**0.007** ^d^	**0.009** ^e^	0.039^e^	NS^e^
Follow‐up (months)	75.5 (54.1–90.3)	75.4 (54.2–90.1)	71.3 (45.6–85.3)	79.3 (50.8–92.3)	NS^d^	—	—	—
Non‐HCC death (*N*, %)	109 (7.1)	82 (6.7)	8 (7.7)	19 (9.3)	NS^c^	—	—	—
Non‐HCC OLT (*N*, %)	1 (0.06)	0 (0)	0 (0)	1 (0.5)	NS^c^	—	—	—
HCC (*N*, %)	50 (3.3)	34 (2.8)	12 (11.5)	4 (2.0)	**< 0.001** ^c^	**< 0.001** ^c^	NS^c^	**0.001** ^c^

*Note:* Quantitative variables are presented as median with first and third quartiles (Q1–Q3) and categorical variables as number and percentage. Comparisons across the three groups are shown in the column ‘Global *p*‐value’; when statistically significant, pairwise comparisons are reported with Bonferroni correction.

Abbreviations: ALBI, albumin–bilirubin score; Alco, alcohol consumption; ALT, alanine aminotransferase; APRI, AST to platelet ratio index; AST, aspartate aminotransferase; BMI, body mass index; CSPH, clinically significant portal hypertension; GGT, gamma‐glutamyl transpeptidase; HCC, hepatocellular carcinoma; INR, international normalised ratio; MELD, Model for End‐Stage Liver Disease; Met, metformin; No‐met, no metformin; NSBB, non‐selective beta‐blocker; OLT, orthotopic liver transplantation; T2DM, type 2 diabetes mellitus.

^a^ Metformin exposure status was defined according to whether patients contributed metformin‐exposed person‐time during follow‐up and is shown for descriptive purposes. Adjusted models used cumulative, time‐updated metformin exposure. Hepatocellular carcinoma (HCC) occurrence is presented as an unadjusted crude proportion and does not account for differences in follow‐up time or time‐varying exposure; adjusted risk estimates were derived from weighted Cox proportional hazards models using cumulative, time‐updated metformin exposure. Percentages were calculated using available data for each variable; denominators may therefore vary slightly across rows because of missing values.

^b^ Haemoglobin A1c level at diagnosis of T2DM. Significant *p*‐values are shown in bold.

^c^ Pearson's chi‐square test.

^d^ Kruskal–Wallis test.

^e^ Mann–Whitney *U* test.

### 3.2 Crude Incidence Rate of Hepatocellular Carcinoma According to FIB‐4 Level, CSPH and Metformin Exposure

**FIGURE 2:**
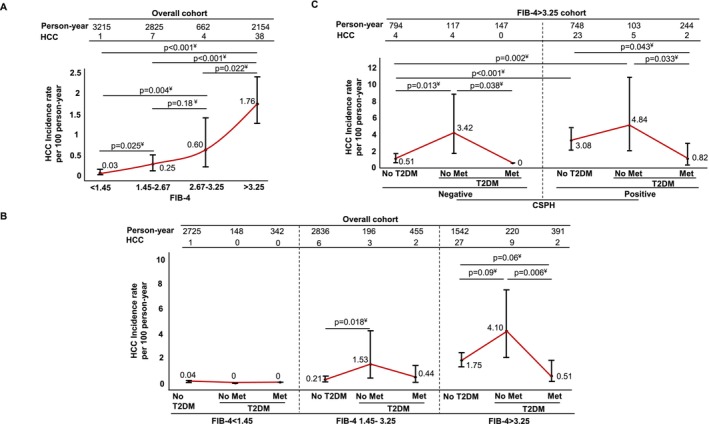
Crude incidence of hepatocellular carcinoma after sustained virologic response, stratified by fibrosis severity, type 2 diabetes mellitus, metformin exposure and clinically significant portal hypertension. Incidence‐rate analyses were conducted among 1529 patients with evaluable baseline FIB‐4 and follow‐up data. (A) Incidence rates of hepatocellular carcinoma according to baseline FIB‐4 categories. (B) Incidence rates according to FIB‐4 category and type 2 diabetes mellitus (T2DM) status, with patients with T2DM further stratified by metformin exposure during follow‐up. (C) Incidence rates among patients with advanced fibrosis (FIB‐4 > 3.25), stratified by clinically significant portal hypertension (CSPH), T2DM status and metformin exposure during follow‐up. Incidence rates are expressed as cases per 100 person‐years. Error bars represent exact Poisson 95% confidence intervals. Comparisons are crude and descriptive; formal adjusted relative association estimates are provided by the weighted Cox models. CSPH, clinically significant portal hypertension; HCC, hepatocellular carcinoma; SVR, sustained virologic response; T2DM, type 2 diabetes mellitus. ^¥^Mid‐P exact test for crude rate comparisons based on event counts and person‐time.

After a median follow‐up of 75 months (Q1: 54; Q3: 90), HCC events were strongly concentrated among patients with higher baseline FIB‐4 values. Incidence‐rate analyses stratified by FIB‐4 were conducted among 1529 patients with evaluable baseline FIB‐4 and follow‐up data. HCC occurred in 1 of 543 patients (0.2%) with FIB‐4 < 1.45, 11 of 599 patients (1.8%) with FIB‐4 1.45–3.25 and 38 of 387 patients (9.8%) with FIB‐4 > 3.25. Accordingly, the crude HCC incidence rate was extremely low among patients with FIB‐4 < 1.45 (0.03 per 100 person‐years; 95% CI: 0.001–0.17), increased modestly in patients with FIB‐4 1.45–3.25 (0.32 per 100 person‐years; 95% CI: 0.16–0.56) and increased sharply in patients with FIB‐4 > 3.25, reaching 1.76 per 100 person‐years (95% CI: 1.25–2.42). When the intermediate FIB‐4 range was further subdivided, incidence rates remained low among patients with FIB‐4 values of 1.45–2.67 and 2.67–3.25, at 0.25 and 0.60 per 100 person‐years, respectively (Figure 2A).

T2DM was associated with a higher crude incidence of HCC within the intermediate and high FIB‐4 strata. Among patients with FIB‐4 1.45–3.25, crude HCC incidence rates were 0.21, 0.44 and 1.53 per 100 person‐years in patients without T2DM, patients with T2DM exposed to metformin and patients with T2DM not exposed to metformin during follow‐up, respectively. A similar pattern was observed among patients with FIB‐4 > 3.25, with corresponding incidence rates of 1.75, 0.51 and 4.10 per 100 person‐years in the same T2DM strata (Figure 2B).

In the subgroup of patients with FIB‐4 > 3.25, CSPH further stratified HCC risk. In the absence of CSPH, crude HCC incidence was 0.50 per 100 person‐years among patients without T2DM and 3.42 per 100 person‐years among patients with T2DM not exposed to metformin, whereas no HCC events were observed among metformin‐exposed patients with T2DM. Conversely, patients with CSPH exhibited a high crude HCC incidence rate of approximately 3.1 per 100 person‐years even in the absence of T2DM. Among patients with both CSPH and T2DM, crude incidence increased in those not exposed to metformin (4.84 per 100 person‐years), whereas metformin‐exposed patients showed a lower incidence rate of 0.82 per 100 person‐years (Figure 2C). These crude descriptive analyses were consistent with the concentration of HCC risk in patients with advanced fibrosis, CSPH and T2DM; formal adjusted relative association estimates are provided by the weighted Cox models.

### 3.3 Predictors of Hepatocellular Carcinoma After SVR and Effect of CME

Because our objective was etiologic inference, cause‐specific hazard models were preferred. During the follow‐up period, 50 patients developed HCC. In the overall weighted multivariable Cox PH model—stratified by sex and incorporating IPTW and IPCW—CME, T2DM, CSPH, FIB‐4 > 3.25 and active smoking status were independently associated with HCC risk. The overall weighted model was fitted in the complete‐case analytic cohort comprising 1489 patients, represented by 1644 start–stop intervals, with 50 HCC events (Figure 1). For transparency, Table S2A reports unweighted and weighted HCC event counts according to metformin exposure during follow‐up, based on the time‐updated exposure definition used in the Cox models.

#### 3.3.1 Independent Predictors of HCC

CSPH was a major independent predictor of incident HCC, conferring more than a sixfold increase in risk (Hazard Ratio (HR) 6.57, 95% CI: 2.21–19.57; *p* < 0.001). Regarding fibrosis severity, FIB‐4 showed a graded association with HCC risk. Patients with FIB‐4 values between 1.45 and 3.25 showed a marked, although not statistically significant, tendency towards increased risk (HR 7.59, 95% CI: 0.89–64.51; *p* = 0.063), whereas those with FIB‐4 > 3.25 showed a significant increase in HCC risk, albeit with a wide confidence interval (HR 15.54, 95% CI: 1.41–171.65; *p* = 0.025). Active smoking was independently associated with HCC development (HR 2.87, 95% CI: 1.32–6.25; *p* = 0.008). In addition, patients with T2DM had an approximately 2.4‐fold higher risk of HCC than non‐diabetic individuals (HR 2.38, 95% CI: 1.16–4.89; *p* = 0.018).

**FIGURE 3:**
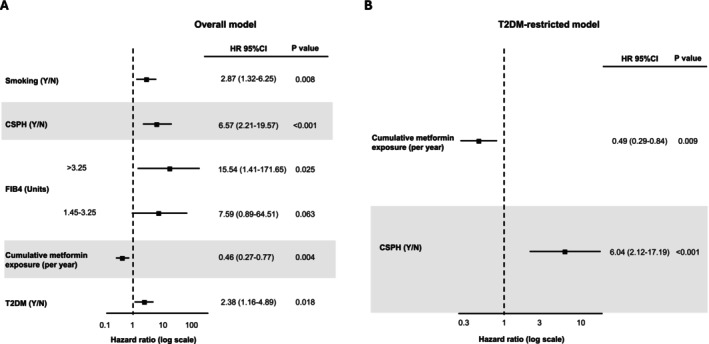
Forest plot of the sex‐stratified multivariable Cox proportional hazards model. (A) Adjusted hazard ratios (HRs) and 95% confidence intervals for the overall cohort model, including cumulative metformin exposure (CME) (per year of exposure), type 2 diabetes mellitus (T2DM), clinically significant portal hypertension (CSPH), FIB‐4 categories and active smoking. (B) Adjusted HRs for the prespecified T2DM‐restricted model, including CME and CSPH. Both models were fitted using stabilised inverse probability of treatment and censoring weights (IPTW × IPCW). HRs > 1 indicate increased risk of hepatocellular carcinoma (HCC), whereas HRs < 1 indicate reduced risk. The proportional hazards assumption was assessed using Schoenfeld residuals (Figure S4). CSPH, clinically significant portal hypertension; HR, hazard ratio; T2DM, type 2 diabetes mellitus; Y/N, yes/no.

After weighting and adjustment for confounding by indication and informative censoring, CME was independently associated with a significant reduction in HCC risk (HR 0.46 per year of cumulative exposure, 95% CI: 0.27–0.77; *p* = 0.004). Because all metformin‐exposed patients had T2DM, causal interpretation of the metformin association was further examined in the prespecified T2DM‐restricted weighted analysis described below. The complete model estimates are provided in Table 2A and a graphical summary of the HRs is shown in Figure 3A.

**TABLE 2:** Weighted Cox proportional hazards models for hepatocellular carcinoma after sustained virologic response.

Panel A. Overall cohort weighted Cox model				
Variable	Coefficient	HR	95% CI	*p*
Cumulative metformin exposure, per year	−0.788	0.46	0.27–0.77	0.004
CSPH	1.882	6.57	2.21–19.57	< 0.001
T2DM	0.868	2.38	1.16–4.89	0.018
FIB‐4 1.45–3.25	2.027	7.59	0.89–64.51	0.063
FIB‐4 > 3.25	2.743	15.54	1.41–171.65	0.025
Active smoking	1.054	2.87	1.32–6.25	0.008

Panel B. T2DM‐restricted weighted Cox model				
Variable	Coefficient	HR	95% CI	*p*
Cumulative metformin exposure, per year	−0.710	0.49	0.29–0.84	0.009
CSPH	1.799	6.04	2.12–17.19	< 0.001

*Note:* Panel A: Model performance: Concordance index = 0.88 (SE 0.018). Likelihood ratio test *p* < 0.001; Wald test *p* < 0.001; score (logrank) test *p* < 0.001; robust score test *p* < 0.001. Panel B: Model performance: Concordance index = 0.83 (SE 0.063). Likelihood ratio test *p* < 0.001; Wald test *p* < 0.001; score (logrank) test *p* < 0.001; robust score test *p* = 0.003. Hazard ratios correspond to weighted start–stop Cox proportional hazards models stratified by sex and fitted with robust variance estimation accounting for repeated intervals per patient. In both models, cumulative metformin exposure was modelled as a time‐updated variable expressed per additional year of exposure. Panel A represents the overall cohort model and incorporates stabilised inverse probability of treatment and censoring weights (IPTW × IPCW). The model was fitted in 1489 patients, represented by 1644 start–stop risk intervals, with 50 HCC events and included cumulative metformin exposure, clinically significant portal hypertension (CSPH), type 2 diabetes mellitus (T2DM), FIB‐4 category and active smoking. Panel B represents the prespecified T2DM‐restricted model used for causal interpretation of the metformin association. The model was fitted in 307 patients, represented by 462 start–stop risk intervals, with 16 HCC events. In this model, stabilised IPTWs were re‐estimated within the T2DM cohort and combined with stabilised IPCWs; the outcome model was adjusted for CSPH.

Abbreviations: CI, confidence interval; CSPH, clinically significant portal hypertension; FIB‐4, fibrosis‐4 index; HR, hazard ratio; IPCW, inverse probability of censoring weighting; IPTW, inverse probability of treatment weighting; SE, standard error; T2DM, type 2 diabetes mellitus.

In the subgroup with baseline LSM available (1282 patients; 35 HCC events), the inverse association between CME and HCC risk remained consistent when FIB‐4 was replaced by LSM (HR 0.53, 95% CI 0.33–0.85; *p* = 0.008). Results were likewise preserved when clinical portal hypertension was replaced by the Baveno VII liver stiffness‐based rule‐in criterion for CSPH, defined as LSM ≥ 25 kPa (HR 0.52, 95% CI 0.31–0.89; *p* = 0.017). CME estimates are provided in Table S4.

### 3.4 Causal Analysis Restricted to Patients With T2DM

In the T2DM‐restricted analytic cohort comprising 307 patients, 462 start–stop risk intervals and 16 HCC events (Figure 1, Table S2B), each additional year of CME was independently associated with a significantly lower hazard of HCC development (HR 0.49; 95% CI 0.29–0.84; *p* = 0.009). Conversely, the presence of CSPH was associated with a markedly increased HCC hazard (HR 6.04; 95% CI 2.12–17.19; *p* < 0.001). The T2DM‐restricted model also showed good apparent discrimination, with a concordance index of 0.83 (SE = 0.063). These findings support the consistency of the inverse association between CME and HCC risk within the clinically relevant T2DM population. The complete model estimates are provided in Table 2B and a graphical summary of the HRs is shown in Figure 3B.

Modelling exposure as a time‐updated covariate ensured correct classification of unexposed and exposed person‐time and avoided immortal‐time bias. The estimated coefficient therefore reflects the average association with HCC risk per year of cumulative exposure. Under the log‐linear specification of the model, longer cumulative exposure was associated with lower predicted HCC risk within the observed exposure range. This estimate should be interpreted as an average association rather than as a constant multiplicative risk reduction extending indefinitely over time.

A sensitivity analysis of weight truncation was performed in the overall and T2DM‐restricted models, indicating that the estimated association between CME and lower HCC risk was not driven by extreme analytic weights (Details are shown in the Supporting Information).

### 3.5 Proportional Hazards Assumption and Performance of the Weighted Cox Models

The proportional hazards assumption was not violated in either the weighted overall model or the T2DM‐restricted model. Both models showed good apparent discrimination, with concordance indices of 0.88 and 0.83, respectively. Detailed Schoenfeld residual diagnostics and model‐performance results are provided in the Supporting Information and Figure S4.

### 3.6 Fine‐Gray Sensitivity Analysis

**FIGURE 4:**
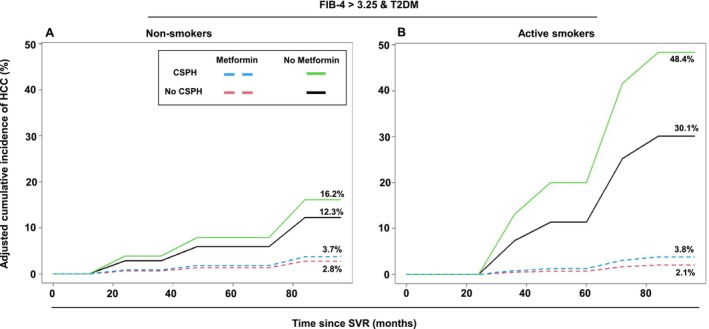
Adjusted cumulative incidence of hepatocellular carcinoma after sustained virological response, derived from multivariable Fine–Gray competing risks regression in patients with type 2 diabetes mellitus and advanced fibrosis (FIB‐4 > 3.25). Curves depict model‐based predictions for a fixed covariate profile, stratified by clinically significant portal hypertension status (absent vs. present) and metformin exposure (no vs. yes), yielding four clinical scenarios. Results are shown separately for non‐smokers (A) and active smokers (B). Curves are shown for illustrative purposes and do not represent formal statistical comparisons; statistical inference is based on subdistribution hazard ratios derived from the Fine–Gray model and is reported in Table S5. Percentages displayed adjacent to the curves indicate the model‐based estimated cumulative incidence of hepatocellular carcinoma at the end of follow‐up. CSPH, clinically significant portal hypertension; HCC, hepatocellular carcinoma; SVR, sustained virological response; T2DM, type 2 diabetes mellitus.

In Fine–Gray models accounting for competing non‐HCC mortality and non‐HCC OLT before HCC occurrence, post‐SVR ever metformin use was associated with a markedly lower cumulative incidence of HCC (subdistribution HR 0.12, 95% CI 0.04–0.37; *p* < 0.001). CSPH, advanced fibrosis, T2DM and active smoking were independently associated with a higher cumulative incidence of HCC in the overall cohort model (Table S5A). Results were directionally consistent in analyses restricted to patients with T2DM, although precision was reduced because of the lower number of events (Table S5B). Figure 4 shows the adjusted cumulative incidence of HCC after SVR, derived from the Fine–Gray competing risks regression in patients with T2DM and advanced fibrosis (FIB‐4 > 3.25), according to CSPH status, metformin exposure and smoking status.

### 3.7 Risk Factors for Hepatocellular Carcinoma Identified by Random Survival Forest Analysis: Role of Metformin

As a complementary, exploratory approach, RSF models with SHAP interpretation were used to examine nonlinear risk patterns and interactions among predictors of HCC. These analyses were prediction‐oriented and were not intended for causal inference.

**FIGURE 5:**
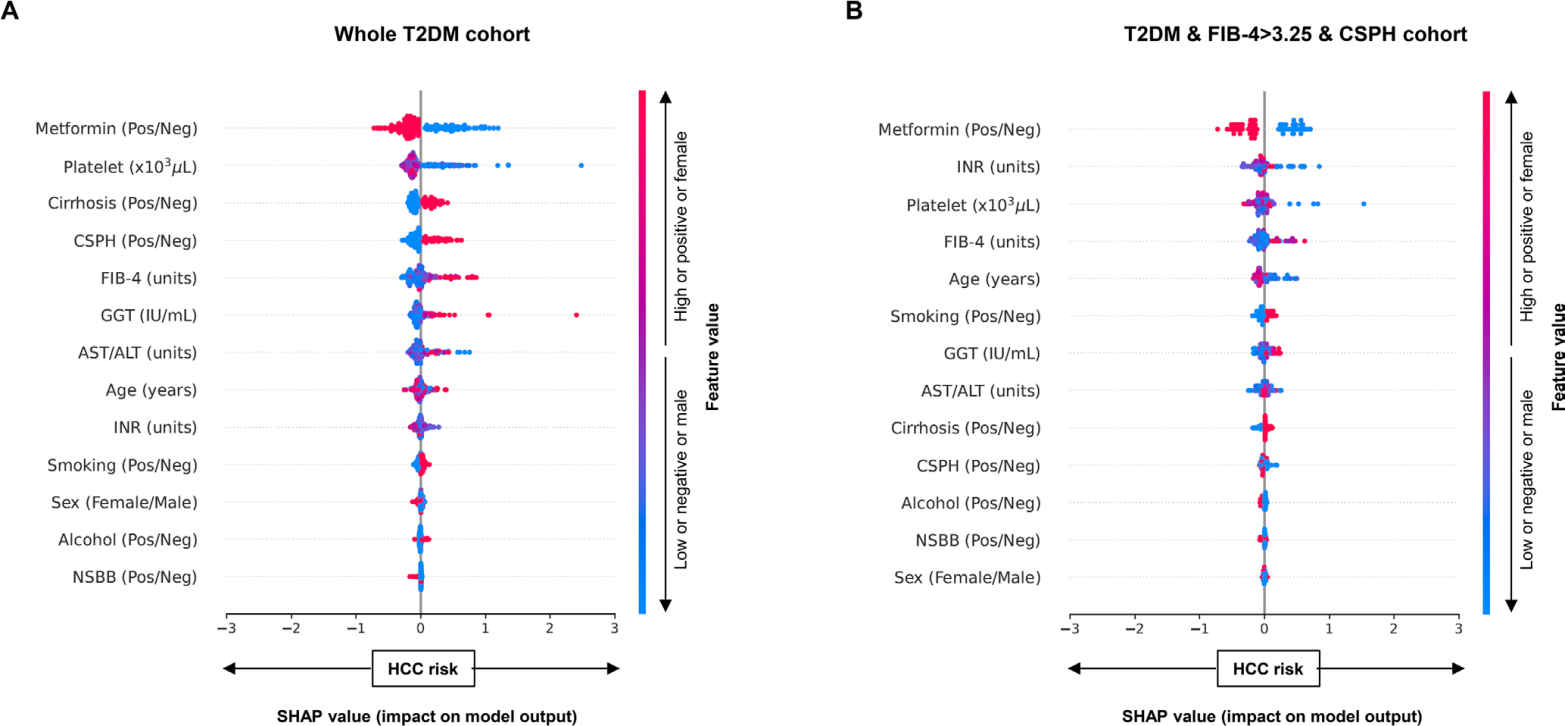
Random survival forest analysis of risk factors for hepatocellular carcinoma restricted to patients with type 2 diabetes mellitus. SHapley Additive exPlanations (SHAP) summary plots depicting the relative importance and direction of baseline covariates for hepatocellular carcinoma (HCC) risk prediction in patients with T2DM. (A) Analysis of the overall T2DM cohort, in which metformin exposure emerged as one of the main contributors towards lower predicted HCC risk. (B) Subgroup analysis of patients with advanced fibrosis (FIB‐4 > 3.25) and clinically significant portal hypertension (CSPH), showing a more pronounced contribution of metformin towards lower predicted HCC risk. Each dot represents an individual patient and the colour denotes the value of the corresponding feature; for binary variables, red indicates higher values or presence of the characteristic and blue indicates lower values or absence. For sex, red indicates female and blue indicates male. SHAP values on the *x*‐axis indicate the magnitude and direction of each variable's contribution to the predicted HCC risk. These plots are exploratory and should not be interpreted as causal effect estimates. ALT, alanine aminotransferase; AST, aspartate aminotransferase; CSPH, clinically significant portal hypertension; GGT, gamma‐glutamyl transpeptidase; HCC, hepatocellular carcinoma; INR, international normalised ratio; NSBB, non‐selective beta‐blocker; SHAP, SHapley Additive exPlanations; T2DM, type 2 diabetes mellitus.

In analyses restricted to patients with T2DM, metformin emerged as a major contributor towards lower predicted HCC risk (Figure 5A). This contribution was most evident among patients with both advanced fibrosis (FIB‐4 > 3.25) and CSPH (Figure 5B). These findings should be interpreted as exploratory predictive signals, since SHAP values quantify contributions to model prediction rather than causal effect magnitude.

Additional RSF analyses in the overall cohort and fibrosis‐defined subgroups, cross‐validation performance metrics and exploratory model‐based exposure contrasts are provided in the Supporting Information and Figures S5 and S6.

## 4 Discussion

In patients with CHC who achieve SVR, the incidence of HCC decreases substantially. Nevertheless, a clinically meaningful residual risk persists and must be accurately defined to optimise preventive strategies and surveillance protocols [1]. In our multicentre cohort, HCC incidence stratified by fibrosis stage closely mirrored that reported in a large meta‐analysis that includes more than 25 000 patients [2]. The FIB‐4 index proved effective for post‐SVR risk stratification, with FIB‐4 > 3.25 identifying the subgroup at the highest risk, consistent with prior evidence [4]. Importantly, however, risk within this category was heterogeneous: patients with FIB‐4 > 3.25 but without T2DM or CSPH had comparatively low incidence rates, whereas the presence of CSPH—particularly in combination with T2DM—was associated with a marked amplification of HCC risk, underscoring the need for risk stratification beyond fibrosis alone [20].

Within this framework, CME—modelled as a time‐updated variable in a sex‐stratified Cox PH model and weighted using stabilised IPTW and IPCW—was independently associated with lower HCC risk after SVR. Importantly, because all metformin‐exposed individuals had T2DM, the clinically relevant causal interpretation of the metformin association was based on the prespecified T2DM‐restricted weighted Cox model, in which the inverse association persisted after recalibration of treatment weights within the metformin‐eligible population. Because daily dose data were unavailable, exposure was modelled as duration rather than intensity, an approach consistent with pharmacoepidemiologic practice when cumulative biological effects are hypothesised [21]. Although metformin exposure was reconstructed from prescription start and stop dates—serving as a proxy for adherence—any resulting misclassification is unlikely to fully explain the magnitude and consistency of the observed association. Although the PH assumption was not violated, this does not necessarily imply that the association between CME and HCC risk is strictly linear over time. Therefore, the HR per additional year of exposure should be interpreted as an average inverse association within the observed exposure range. A nonlinear exposure–response relationship, including a possible plateau effect, cannot be excluded.

Our results are consistent with prior observational studies reporting a reduced HCC risk among metformin‐treated patients with T2DM [6, 7, 11, 22]. Tsai et al. [6] demonstrated a lower post‐SVR HCC incidence in diabetic patients receiving metformin, whereas Tseng et al. [7] reported a clear inverse association with cumulative exposure duration across increasing tertiles of cumulative exposure. Although our continuous time‐updated model is not directly comparable with these categorical approaches, the concordant direction of association supports the external consistency of a cumulative exposure signal across populations and analytical frameworks [23].

Beyond metformin, our findings reaffirm the central role of CSPH, advanced fibrosis, T2DM and active smoking as the major determinants of post‐SVR HCC risk [2, 24]. The competing‐risk analysis supported the direction of the primary findings, suggesting that the inverse association was not explained by differential non‐HCC mortality or OLT. Because Fine–Gray models required simplification of metformin exposure to post‐SVR ever use, the resulting subdistribution HR was interpreted only as supportive directional evidence and was not compared numerically with the primary cumulative‐exposure HR.

Machine‐learning analyses using RSF and SHAP [18, 19] provided complementary exploratory evidence, highlighting advanced liver disease as the dominant predictive domain and suggesting that metformin contributed towards lower predicted HCC risk within clinically high‐risk T2DM subgroups.

The biological plausibility of metformin's inverse association with HCC risk is supported by its activation of AMPK and inhibition of oncogenic signalling pathways such as PI3K/Akt/mTORC1 [8, 9, 10, 25], as well as potential immunometabolic effects that enhance tumour surveillance [9, 25]. However, in advanced liver disease, irreversible architectural distortion and a pro‐oncogenic microenvironment may partially offset these effects, contributing to risk heterogeneity [26].

This study has several strengths, including its multicentre design, long follow‐up duration, detailed ascertainment of metformin exposure using start‐ and stop‐dates and integration of advanced causal inference methods combining IPTW and IPCW. Limitations include the observational nature of the study, absence of detailed information on metformin dose and adherence and lack of data on aspirin exposure, which may also influence HCC risk. Post‐SVR HCC surveillance was not standardised in patients without advanced fibrosis, although HCC events and vital status were ascertained through electronic health records and telephone contact when required, making major underascertainment unlikely. The limited number of HCC events may also have reduced the precision of some hazard ratio estimates, particularly for covariates with sparse subgroup categories such as FIB‐4 and therefore effect sizes should be interpreted cautiously. The limited number of events also constrained formal assessment of non‐linear cumulative exposure–response patterns; therefore, a threshold or plateau effect of metformin cannot be excluded. CSPH was defined using pragmatic surrogate markers routinely used in clinical practice, because baseline liver transient elastography values were not available for all patients, particularly those recruited in the earlier years; therefore, current Baveno VII elastography‐based criteria could not be applied uniformly and some non‐differential misclassification cannot be excluded. Nevertheless, sensitivity analyses in the subgroup with available LSMs yielded consistent estimates when FIB‐4 was replaced by LSM and when the pragmatic CSPH definition was replaced by the Baveno VII LSM‐based rule‐in criterion for CSPH, defined as LSM ≥ 25 kPa. Because IPTW covariates were measured only at baseline, time‐varying confounding related to evolving liver status or diabetes management during follow‐up cannot be excluded. Residual confounding related to metformin eligibility, intolerance, frailty, renal‐function trajectories or clinician avoidance in sicker patients cannot be excluded. Future studies should explore combined chemopreventive strategies incorporating pharmacological and lifestyle interventions, such as statins, NSAIDs, smoking cessation and alcohol reduction [24].

In conclusion, FIB‐4 remains a robust tool for post‐SVR HCC risk stratification and is further refined by smoking, CSPH and T2DM status. In an IPTW–IPCW weighted Cox PH model incorporating cumulative time‐dependent exposure, metformin was associated with lower HCC risk, with causal interpretation restricted to patients with T2DM. These findings support further evaluation of metformin as a potential adjunctive chemopreventive strategy that should complement—rather than replace—structured HCC surveillance.

## Author Contributions

Conceptualisation: Juan‐Ramón Larrubia and Conrado Fernández‐Rodríguez. Methodology: Juan‐Ramón Larrubia, Conrado Fernández‐Rodríguez, Miguel Torralba and Óscar Barquero‐Pérez. Machine learning analysis (RSF, SHAP): Óscar Barquero‐Pérez, Myriam Catalá and José Gómez. Investigation/data collection: Henar Calvo‐Sánchez, Lorena Jara‐Fernández, Sonia Albertos, Raquel Encijo‐Heredia, Rubén Alvarado, Irene Villarino, Marta Quiñones‐Calvo, María‐Luisa Gutiérrez and Joaquín Miquel. Data curation and formal analysis: Juan‐Ramón Larrubia, Conrado Fernández‐Rodríguez, Henar Calvo‐Sánchez, Lorena Jara‐Fernández and Óscar Barquero‐Pérez. Funding acquisition: Juan‐Ramón Larrubia, Óscar Barquero‐Pérez. Writing – original draft: Juan‐Ramón Larrubia, Óscar Barquero‐Pérez and Conrado Fernández‐Rodríguez. Writing – review and editing: All authors. Guarantor: Juan‐Ramón Larrubia. Final approval: All authors.

## Funding

This work was partially funded by the Instituto de Salud Carlos III (ISCIII), Spain, through the State Plan for Scientific and Technical Research and Innovation 2013–2016, grant PI15/00074 (JRL), co‐funded by ERDF—European Union. Additional support was provided by grant PID2022‐136887NB‐I00, funded by MCIN/AEI/10.13039/501100011033 (OBP).

## Ethics Statement

Approved by the Research Ethics Committee of Guadalajara University Hospital, Spain (Approval No. ACTA N1/2023), with adherence by all participating centres.

## Consent

Due to the retrospective nature of the study and use of anonymised data, informed consent was waived by the ethics committees of all participating institutions.

## Conflicts of Interest

The authors declare no conflicts of interest.

## Supporting information


**Figure S1:** Covariate balance before and after stabilised inverse probability of treatment weighting. (A) Absolute standardised mean differences (SMDs) for the covariates included in the propensity score model used to derive stabilised IPTWs in the overall cohort. (B) The corresponding balance diagnostics for the propensity score model used to derive stabilised IPTWs in the T2DM‐restricted cohort. Red circles represent unweighted estimates and green circles represent weighted estimates. The green‐shaded area indicates adequate covariate balance, defined as an absolute SMD between 0 and 0.10. After weighting, all covariates included in both propensity score models were within the prespecified balance threshold.
**Figure S2:** Distribution of inverse probability of censoring weights (IPCW) and combined analytic weights. (A) The distribution of stabilised IPCW derived from the overall censoring model. (B) The distribution of total weights used in the overall weighted Cox model, derived as the product of overall stabilised inverse probability of treatment and censoring weights (IPTW × IPCW). (C) The distribution of total weights used in the T2DM‐restricted weighted Cox model, calculated as the product of T2DM‐specific stabilised IPTW and the overall stabilised IPCW. Across panels, weight distributions were compact and well behaved, with no evidence of highly influential extreme values and only a limited right tail for the combined weights, supporting the stability of the weighting approach used in the weighted Cox proportional hazards analyses.
**Figure S3:** Marginal non‐censoring survival function, G(t), estimated from the overall censoring model. G(t) represents the probability of remaining uncensored over time, with censoring defined as loss to follow‐up, non‐HCC death or non‐HCC OLTbefore HCC occurrence. The resulting IPCW were used in both the overall and T2DM‐restricted weighted Cox analyses. G(t) remained above 0.80 until approximately 2500 days of follow‐up.
**Figure S4:** Schoenfeld residual diagnostics for the weighted multivariable Cox proportional hazards models. (A) The Schoenfeld residual plots and corresponding global and covariate‐specific test results for the weighted overall model. (B) The analogous diagnostics for the T2DM‐restricted model. In both models, neither the global nor the covariate‐specific Schoenfeld tests provided evidence of violation of the proportional hazards assumption, and visual inspection of the residual plots showed no clear sustained time‐dependent trends. CSPH, clinically significant portal hypertension; T2DM, type 2 diabetes mellitus. ^†^Chi‐square test.
**Figure S5:** Random survival forest analysis of risk factors for hepatocellular carcinoma. SHapley Additive exPlanations (SHAP) summary plots showing the relative importance and direction of baseline covariates in (A) the overall cohort, (B) the high‐risk subgroup with FIB‐4 > 3.25 and clinically significant portal hypertension and (C) the subgroup with FIB‐4 > 3.25 without clinically significant portal hypertension. Each dot represents an individual patient, and the colour denotes the value of the corresponding feature; for binary variables, red indicates higher values or presence of the characteristic and blue indicates lower values or absence. For sex, red indicates female and blue indicates male. SHAP values on the x‐axis indicate the magnitude and direction of each variable's contribution to the predicted risk of hepatocellular carcinoma. ALT, alanine aminotransferase; AST, aspartate aminotransferase; CSPH, clinically significant portal hypertension; GGT, gamma‐glutamyl transpeptidase; HCC, hepatocellular carcinoma; INR, international normalised ratio; NSBB, non‐selective beta‐blocker; T2DM, type 2 diabetes mellitus.
**Figure S6:** Estimated probability density functions of model‐based average risk contrasts associated with metformin exposure. Distributions represent differences in predicted hepatocellular carcinoma (HCC) risk derived from Random Survival Forest–based model predictions under hypothetical metformin exposure versus non‐exposure scenarios. These contrasts are exploratory, population‐specific and model‐dependent and are intended to provide supportive insights rather than causal or confirmatory inference. Distributions are shown for the overall cohort (blue curve) and for subgroups defined by advanced fibrosis (FIB‐4 > 3.25) without clinically significant portal hypertension (CSPH) (red curve) and with CSPH (green curve) and, separately for (A) the entire study population and (B) patients with type 2 diabetes mellitus (T2DM). Negative values indicate lower model‐predicted HCC risk associated with metformin exposure. CSPH = 0, absence of clinically significant portal hypertension; CSPH = 1, presence of clinically significant portal hypertension; T2DM, type 2 diabetes mellitus.
**Table S1:** Concordance between the study‐defined pragmatic CSPH variable and the Baveno VII liver stiffness‐based rule‐in criterion for clinically significant portal hypertension.
**Table S2:** Unweighted and weighted numbers of hepatocellular carcinoma events according to metformin exposure status in the weighted Cox models.
**Table S3:** Distribution of antidiabetic treatments among T2DM according to metformin exposure status during follow‐up.
**Table S4:** Sensitivity analyses replacing FIB‐4 with liver stiffness measurement and clinical portal hypertension with the Baveno VII liver stiffness‐based rule‐in criterion for CSPH.
**Table S5:** Fine–Gray competing‐risk regression models for hepatocellular carcinoma after sustained virologic response.

## Data Availability

Individual‐level data cannot be publicly shared due to institutional and data‐protection restrictions; anonymised summary‐level data and statistical analysis code may be available upon reasonable request, subject to ethics committee approval.
