# Supplementary material for: Cumulative Metformin Use and Hepatocellular Carcinoma Risk After HCV SVR: A Multicentre Cohort Study
Source: Liver Int. 2026 Jul 24;46(8):e70798. doi: 10.1111/liv.70798 (PMC13400749; doi:10.1111/liv.70798)
Supplement: Supplementary file 1 — Figure S1: Covariate balance before and after stabilised inverse probability of treatment weighting. (A) Absolute standardised mean differences (SMDs) for the covariates included in the propensity score model used to derive stabilised IPTWs in the overall cohort. (B) The corresponding balance diagnostics for the propensity score model used to derive stabilised IPTWs in the T2DM‐restricted cohort. Red circles represent unweighted estimates and green circles represent weighted estimates. The green‐shaded area indicates adequate covariate balance, defined as an absolute SMD between 0 and 0.10. After weighting, all covariates included in both propensity score models were within the prespecified balance threshold. Figure S2: Distribution of inverse probability of censoring weights (IPCW) and combined analytic weights. (A) The distribution of stabilised IPCW derived from the overall censoring model. (B) The distribution of total weights used in the overall weighted Cox model, derived as the product of overall stabilised inverse probability of treatment and censoring weights (IPTW × IPCW). (C) The distribution of total weights used in the T2DM‐restricted weighted Cox model, calculated as the product of T2DM‐specific stabilised IPTW and the overall stabilised IPCW. Across panels, weight distributions were compact and well behaved, with no evidence of highly influential extreme values and only a limited right tail for the combined weights, supporting the stability of the weighting approach used in the weighted Cox proportional hazards analyses. Figure S3: Marginal non‐censoring survival function, G(t), estimated from the overall censoring model. G(t) represents the probability of remaining uncensored over time, with censoring defined as loss to follow‐up, non‐HCC death or non‐HCC OLTbefore HCC occurrence. The resulting IPCW were used in both the overall and T2DM‐restricted weighted Cox analyses. G(t) remained above 0.80 until approximately 2500 days of follow‐up. Figure S4: [file LIV-46-0-s001.docx]

**Cumulative metformin use and hepatocellular carcinoma risk after HCV SVR: a multicenter cohort study**

Henar Calvo-Sánchez^†^, Lorena Jara-Fernández^†^, Raquel Encijo-Heredia, Irene Villarino, Rubén Alvarado, Marta Quiñones-Calvo, María-Luisa Gutiérrez, Joaquín Miquel, Miguel Torralba, Myriam Catalá, José Gómez, Sonia Albertos, Óscar Barquero-Pérez, Conrado Fernández-Rodríguez^‡^, Juan-Ramón Larrubia^‡^.

**Supplementary Material & Methods** **3**

- Sample Size Estimation
- Study population inclusion/exclusion and follow-up protocol
- Agreement between the pragmatic study-defined CSPH variable and the Baveno VII liver stiffness-based rule-in criterion
- Estimation of inverse probability weights
- IPTW Diagnostics
- Diagnostics of IPCW and Censoring Model
- Multivariable Cox Proportional Hazards Model Performance and events per covariate
- Machine Learning–Based Exploratory Analyses

**Supplementary Results** **12**

- Sensitivity analysis of weight truncation
- Proportional hazards assumption and performance of the weighted Cox models
- Additional RSF–SHAP exploratory analyses
- RSF Model Performance
- Exploratory Model-Based Exposure Contrasts

**Supplementary Figures** **S1-S6** **16**

**Supplementary Tables** **S1-S5** **24**

**References** **32**

**Supporting information**

**Supplementary Methods**

**Sample Size Estimation**

A sample-size adequacy calculation was performed using a feasibility-based approximation derived from the expected incidence of hepatocellular carcinoma (HCC), the anticipated magnitude of the association between metformin exposure and HCC risk, and the proportion of patients expected to receive metformin. Based on published estimates, we assumed that approximately 20% of the cohort would have type 2 diabetes mellitus (T2DM)^1^. We further assumed that approximately 60-80% of these patients would be treated with metformin^2^, corresponding to an overall metformin exposure of approximately 13%.

Schoenfeld’s event-based approximation for Cox proportional hazards models, and under a simplified binary metformin exposure assumption, the study was planned to detect a hazard ratio (HR) of 0.30 for HCC in metformin users compared with non-users. Assuming an overall cumulative HCC incidence of 3.5% over a median follow-up of 75 months, consistent with published post-sustained virological response (SVR) HCC incidence estimates according to fibrosis stage (Ref #2, main text), a two-sided α level of 0.05, and 80% statistical power, approximately 48 HCC events were required, corresponding to a minimum sample size of 1,368 patients.

This calculation was intended as a feasibility-based approximation and should not be interpreted as a formal power calculation specifically tailored to the cumulative time-updated exposure model and weighted Cox framework used in the primary analysis. Sample size calculations were performed using R version 4.4.3 (R Foundation for Statistical Computing, Vienna, Austria).

**Study population inclusion/exclusion and follow-up protocol**

The inclusion criteria were as follows: (1) age ≥20 years, (2) documented HCV RNA positivity for ≥6 months before treatment, (3) treatment with direct-acting antivirals for ≥4 weeks, (4) achievement of SVR, and (5) availability of metformin exposure and post-SVR follow-up information. Exclusion criteria were HBV or HIV coinfection, death before SVR, HCC diagnosed before or within 6 months after SVR, or <6 months of post-SVR follow-up.

Patients were treated and followed between August 2012 and December 2022. Individuals treated before April 2015 received direct-acting antivirals under a national compassionate-use program. After SVR, patients with advanced fibrosis (F3–F4) underwent semiannual HCC surveillance with abdominal ultrasound and α-fetoprotein according to routine clinical practice. Patients with F0–F2 fibrosis were generally discharged from routine hepatology follow-up and did not undergo a standardized HCC surveillance program; however, HCC occurrence and vital status were ascertained throughout follow-up through electronic health records and telephone contact when required. SVR was defined as undetectable HCV RNA 12 weeks after therapy completion. HCC was diagnosed according to EASL guidelines^3^.

**Agreement between the pragmatic study-defined CSPH variable and the Baveno VII liver stiffness-based rule-in criterion**

Among patients with available baseline liver stiffness measurement, concordance between this pragmatic definition and the contemporary Baveno VII liver stiffness-based rule-in criterion for CSPH, defined as liver stiffness measurement ≥25 kPa, was moderate-to-substantial (overall agreement, 92.0%; Cohen’s κ=0.61) (**Supplementary Table S1**). This supports that the study-defined CSPH variable captured a clinically meaningful portal hypertension phenotype, while acknowledging that LSM ≥25 kPa is a highly specific rule-in rather than an exhaustive case-finding criterion.

**Estimation of inverse probability weights**

*Stabilized inverse probability of treatment weights (IPTW) for metformin exposure*: A logistic regression model was used to estimate the individual probability of receiving metformin based on baseline covariates considered clinically relevant for treatment assignment in the overall cohort. The propensity score (PS) model included alcohol consumption, serum albumin level, liver fibrosis stage, use of NSBBs and statins, and renal function assessed by serum creatinine. In the T2DM-restricted cohort, the PS model was re-estimated using the same covariate set as in the overall cohort, plus HbA1c category at T2DM diagnosis and non-metformin glucose-lowering treatment. All covariates used for IPTW estimation were defined at baseline and not updated over time. Stabilized IPTWs were constructed using the marginal probability of metformin treatment and applied to balance baseline covariates between exposed and unexposed individuals. PS models and stabilized weights were estimated among patients with complete data for the covariates included.

*Stabilized inverse probability of censoring (IPCW)*: A Cox proportional hazards (PH) model estimated the probability of remaining alive and uncensored over time. Follow-up began on the date of SVR12. Censoring was defined as loss to follow-up, non-HCC death or non-HCC orthotopic liver transplantation (OLT) before HCC occurrence. Predictors included age, sex, liver cirrhosis, and T2DM, selected a priori because of their potential influence on follow-up completeness and pre-HCC follow-up termination. IPCW derived from the full cohort censoring model were retained for the T2DM-restricted model, as this model included T2DM status and other predictors of censoring, thereby providing conditional estimates of remaining uncensored for T2DM participants. The distribution of these weights was additionally evaluated within the T2DM subgroup.

*Combined weights*: For each risk interval, the final weight applied to the overall and T2DM-restricted weighted Cox PH models was: 𝑊total=𝑊IPTW×𝑊IPCW.

**IPTW Diagnostics**

To assess the adequacy of stabilized IPTW, the PS covariate balance between metformin-exposed and unexposed patients was evaluated using standardized mean differences (SMDs) in both the overall cohort and the T2DM-restricted cohort.

Covariate balance was examined visually using a Love plot, displaying SMDs before and after application of stabilized IPTW for the overall PS model and the T2DM-restricted PS model, respectively. Several covariates exhibited meaningful imbalance prior to weighting, with SMDs exceeding the commonly accepted threshold of 0.10 (**Supplementary Figure S1**).

After the application of stabilized IPTW, all covariates included in both PS models achieved an excellent balance, with every adjusted SMD falling below 0.10. This indicates an effective reduction in measured baseline imbalance between treatment groups and supports adequate covariate balance after weighting (**Supplementary Figure S1**).

**Diagnostics of IPCW and Censoring Model**

We conducted several diagnostics to assess the stability and adequacy of the IPCW and combined weights (IPTW×IPCW) applied in the weighted Cox PH analyses. The IPCW model was treated as a nuisance model for estimating the probability of remaining uncensored, and its adequacy was evaluated primarily through the empirical distribution of the derived weights and the stability of the weighted analyses.

First, we examined the distribution of stabilized IPCW derived from the global censoring model. Summary statistics were as follows: minimum = 0.29, first quartile = 0.69, median = 0.85, mean = 0.80, third quartile = 0.93, and maximum = 1.00. The 5th and 95th percentiles were 0.44 and 0.99, respectively. These values indicate a compact and bounded distribution, with no evidence of extreme or unstable IPCW. Consistently, the histogram of stabilized IPCW (**Supplementary Figure S2A**) showed most weights were concentrated in the upper part of the distribution, indicating that the censoring model introduced only limited weight variability across risk intervals.

Second, we evaluated the distribution of the total weights (IPTW×IPCW) used in the weighted Cox models. In the overall model, combined weights (IPTW×IPCW) had a minimum of 0.07, first quartile of 0.55, median of 1.05, mean of 1.06, third quartile of 1.32, and maximum of 4.53. The upper tail remained moderate, with the 95th and 99th percentiles at 2.25 and 4.05, respectively. The corresponding histogram (**Supplementary Figure S2B**) showed a well-behaved distribution, with most observations clustered around 1 and only a limited right tail.

The distribution of total weights in the T2DM-restricted model, obtained by combining T2DM-specific IPTW with the global IPCW, was similarly well behaved: minimum = 0.18, first quartile = 0.64, median = 0.94, mean = 1.05, third quartile = 1.27, and maximum = 4.62. The 95th and 99th percentiles were 2.17 and 3.42, respectively. Thus, re-estimation of the treatment weights within the T2DM-only cohort did not introduce additional weight instability or a materially more extreme upper tail in the combined weighting scheme (**Supplementary Figure S2C**).

Finally, we examined the marginal non-censoring survival function, G(t), representing the probability of remaining uncensored over time. G(t) remained high for most of the follow-up period and declined below 0.80 only after approximately 2,500 days (≈6.8 years), indicating the probability of remaining uncensored was preserved during most of the clinically relevant observation window. The IPCW used in both the overall and T2DM-restricted weighted Cox models were derived from the same global censoring model; therefore, the marginal non-censoring survival function G(t) applies to both analyses (**Supplementary Figure S3**).

Taken together, the bounded distributions of stabilized IPCW and combined weights, the absence of highly influential extreme values, and the overall stability of weighting structure across global and T2DM-restricted analyses support the practical adequacy and numerical stability of the IPCW approach for the weighted Cox regression analyses.

Weighted event counts are displayed in **Supplementary Table S2** for transparency. They represent the sum of stabilized IPTW×IPCW weights across start–stop intervals in which HCC occurred and therefore reflect pseudo-population contributions to the weighted Cox estimating equations rather than additional observed cases. Patients in **Supplementary Table S2** were classified according to whether they contributed at least one interval with non-zero cumulative metformin exposure during follow-up. This time-updated definition differs from the baseline metformin classification used in **Table 1**, which reflects treatment status during antiviral therapy. Consequently, the number of ever-exposed patients in **Supplementary Table S2** may differ from the baseline metformin count because of differences in exposure accrual during follow-up and the complete-case requirements of both weighted Cox models.

**Multivariable Cox Proportional Hazards Model Performance and events per covariate**

The overall multivariable Cox model included six effective degrees of freedom, with 50 observed HCC events, yielding approximately 8.3 events per effective degree of freedom. Although slightly below the traditional rule of thumb of 10 events per degree of freedom, this ratio was considered reasonable for a pre-specified, clinically parsimonious model and was interpreted with appropriate caution^4^. The T2DM-restricted model included two effective degrees of freedom, with 16 HCC events, yielding 8.0 events per effective degree of freedom. The limited model complexity, use of stabilized weighting, and robust variance estimation were intended to reduce model instability. The concordance indices were 0.88 for the overall model and 0.83 for the T2DM-restricted model, indicating good apparent discriminative ability.

Model performance was evaluated using hazard ratios (HRs) with 95% confidence intervals, Wald and robust score tests, and concordance index. All analyses were performed in R version 4.4.3 using the survival package, (R Foundation for Statistical Computing, Vienna, Austria).

**Machine Learning–Based Exploratory Analyses**

To complement the primary Cox PH analyses, we implemented a Random Survival Forest (RSF) model to explore potential nonlinear effects and higher-order interactions among predictors of HCC. RSF analyses were performed as complementary, prediction-oriented exploratory analyses and were not intended for causal inference. Accordingly, the RSF models were trained using unweighted data.

Model interpretability was assessed using SHapley Additive exPlanations (SHAP), which assign additive feature-attribution values to each covariate for individual model predictions. SHAP values were computed from out-of-fold predictions to reduce information leakage.

To further explore the association of metformin within this framework, counterfactual RSF-based predictions were used to estimate individualized model-based hypothetical exposure contrasts by comparing predicted HCC risk under “metformin-exposed” and “metformin-unexposed” scenarios^5^. These contrasts were considered exploratory predictive quantities and were not interpreted as causal treatment effects. They were intended to provide supportive insights, particularly within high-risk subgroups defined by advanced fibrosis, CSPH, and T2DM.

Hyperparameter optimization was performed using Optuna^6^, and model performance was evaluated by 5-fold cross-validation. All analyses were conducted in Python using Scikit-survival v0.21.0, Scikit-learn v1.2.2, Optuna v3.5.0, and SHAP v0.42.1.

**Supplementary Results**

**Sensitivity analysis of weight truncation**

Sensitivity analyses based on truncation of total analytic weights supported the robustness of the primary findings. In the overall weighted analytic cohort, the HR per additional year of cumulative metformin exposure was 0.455 (95% CI, 0.267–0.775; p=0.0037) using untruncated weights, 0.456 (95% CI, 0.268–0.776; p=0.0038) after truncation at the 1st–99th percentiles, and 0.467 (95% CI, 0.284–0.768; p=0.0027) after truncation at the 5th–95th percentiles. Consistent results were observed in the T2DM-restricted weighted Cox model, where the corresponding HRs were 0.492 (95% CI, 0.288–0.841; p=0.009), 0.492 (95% CI, 0.289–0.837; p=0.009), and 0.502 (95% CI, 0.309–0.816; p=0.005), respectively. These findings indicate that the estimated association between cumulative metformin exposure and lower HCC risk was not driven by extreme analytic weights.

**Proportional hazards assumption and performance of the weighted Cox models**

Neither the global nor the covariate-specific Schoenfeld residual tests showed evidence of violation of the PH assumption in the weighted overall model (global p=0.121; all covariate-specific p>0.05). Similar findings were observed in the T2DM-restricted parsimonious model: the global test was non-significant (p=0.136), with no evidence of non-proportionality for CSPH (p=0.437), whereas cumulative metformin exposure showed a borderline but non-significant result (p=0.066). Individual Schoenfeld residual plots for all model covariates are shown in **Supplementary Figure S4**. The weighted overall and T2DM-restricted Cox models showed high apparent discriminative ability, with a concordance index of 0.88 (SE=0.018) and 0.83 (SE=0.063) respectively. Because formal testing and visual inspection did not indicate a clear sustained temporal trend, no additional time-by-covariate interaction terms were introduced, and conventional weighted PH Cox models were considered appropriate.

**Additional RSF–SHAP exploratory analyses**

In the overall cohort, markers of advanced liver disease—including CSPH, cirrhosis, elevated baseline FIB-4, thrombocytopenia, increased GGT, and higher INR—were the dominant contributors to predicted HCC risk, whereas metformin showed limited overall contribution (**Supplementary Figure S5A**).

When analyses were restricted to patients with advanced fibrosis (FIB-4 >3.25), metformin became more prominent among variables associated with lower predicted HCC risk (**Supplementary Figure S5B**). By contrast, among patients with advanced fibrosis but without CSPH, metformin contributed minimally to RSF prediction and showed no meaningful protective pattern (**Supplementary Figure S5C**). These findings support the interpretation that the predictive relevance of metformin was concentrated in clinically higher-risk strata, while remaining strictly exploratory and non-causal.

**RSF Model Performance**

The RSF demonstrated strong predictive performance, with a concordance index of 0.91 (95% CI: 0.85–0.96) and an AUC of 0.91 (95% CI: 0.86–0.97) across five-fold cross-validation. Performance was consistent across folds, supporting the internal stability of the RSF framework for exploratory assessment of HCC risk patterns. These metrics were not used for confirmatory inference.

**Exploratory Model-Based Exposure Contrasts**

To further explore the association between metformin exposure and HCC risk within the RSF framework, model-based hypothetical exposure contrasts were estimated across predefined risk strata.

In the overall cohort, metformin exposure was associated with a negative model-based average risk contrast (–0.16; 95% CI: –1.28 to 0.05), suggesting a tendency toward lower predicted HCC risk, although this contrast did not reach statistical significance. The corresponding model-based predicted relative risk (RR) estimate was 0.87 (95% CI: 0.85 to 0.90). Among the patients with advanced fibrosis (FIB-4 >3.25), the magnitude of the inverse predictive association increased. In individuals with concomitant CSPH, metformin exposure was associated with a larger reduction in predicted HCC risk (–0.73; 95% CI: –2.33 to –0.01), with a corresponding predicted RR of 0.86 (95% CI: 0.84 to 0.89). In contrast, among patients with advanced fibrosis without CSPH, the estimated effect was smaller and did not reach statistical significance (–0.13; 95% CI: –0.57 to 0.04; predicted RR 0.91; 95% CI: 0.87 to 0.95), (**Supplementary Figure S6A**).

As metformin exposure occurred exclusively among patients with T2DM, a secondary counterfactual analysis was restricted to this subgroup to align the contrast with the metformin-eligible population. Within the T2DM population, metformin exposure was associated with a pronounced reduction in predicted HCC risk (average risk contrast –0.67; 95% CI: –2.28 to –0.02), corresponding to a predicted RR of 0.47 (95% CI 0.30 to 0.64). The strongest inverse predictive association was observed among patients with both advanced fibrosis and CSPH (average risk contrast –1.35; 95% CI: –2.64 to –0.14; predicted RR 0.50; 95% CI 0.33 to 0.67), (**Supplementary Figure S6B**). These model-based contrasts were exploratory and should not be interpreted as causal treatment effects.

**Supplementary Figures**

**
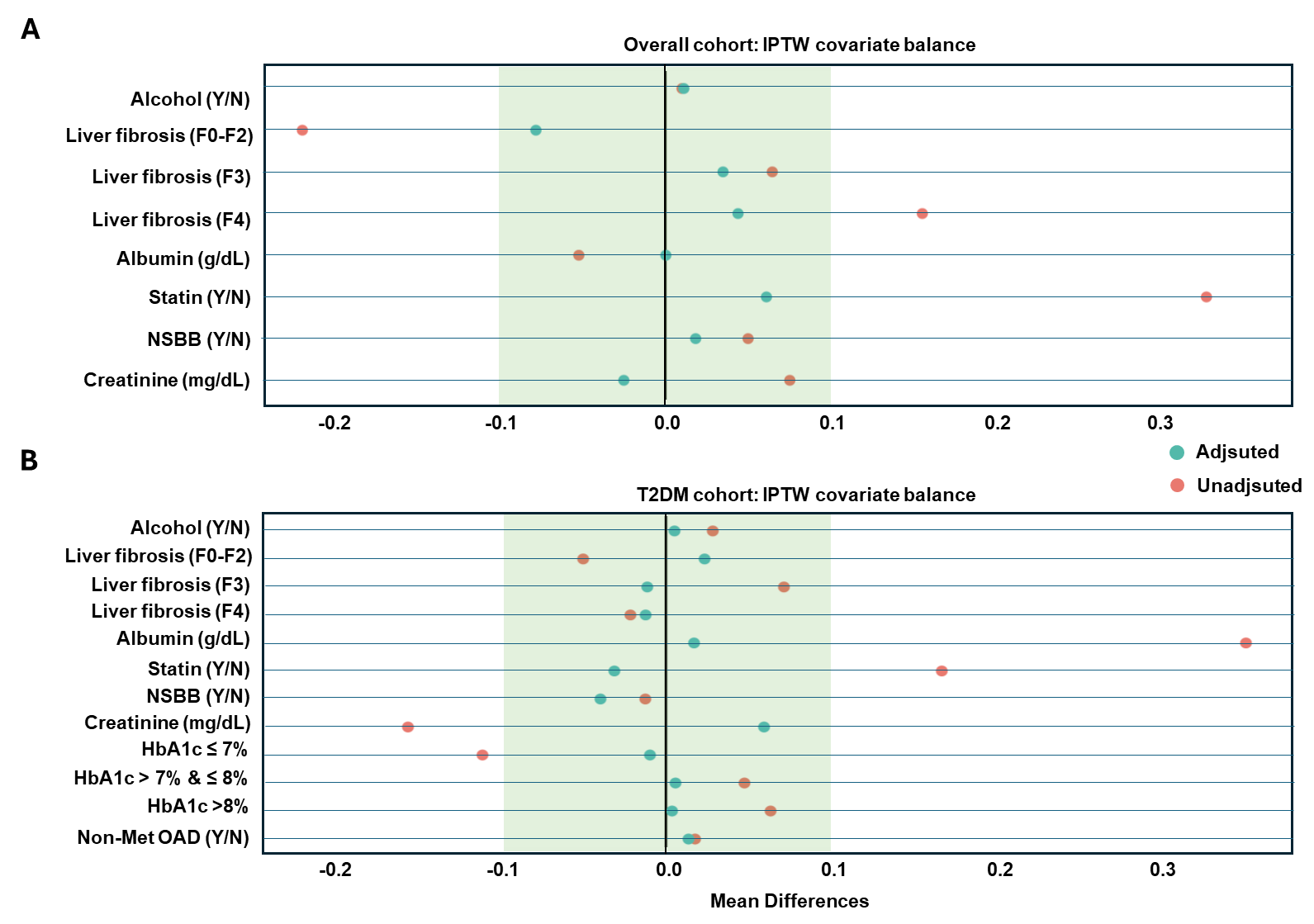
**

**Supplementary Figure S1. Covariate balance before and after stabilized inverse probability of treatment weighting.** Panel (A) shows absolute standardized mean differences (SMDs) for the covariates included in the propensity score model used to derive stabilized IPTWs in the overall cohort. Panel (B) shows the corresponding balance diagnostics for the propensity score model used to derive stabilized IPTWs in the T2DM-restricted cohort. Red circles represent unweighted estimates and green circles represent weighted estimates. The green-shaded area indicates adequate covariate balance, defined as an absolute SMD between 0 and 0.10. After weighting, all covariates included in both propensity score models were within the prespecified balance threshold.

**
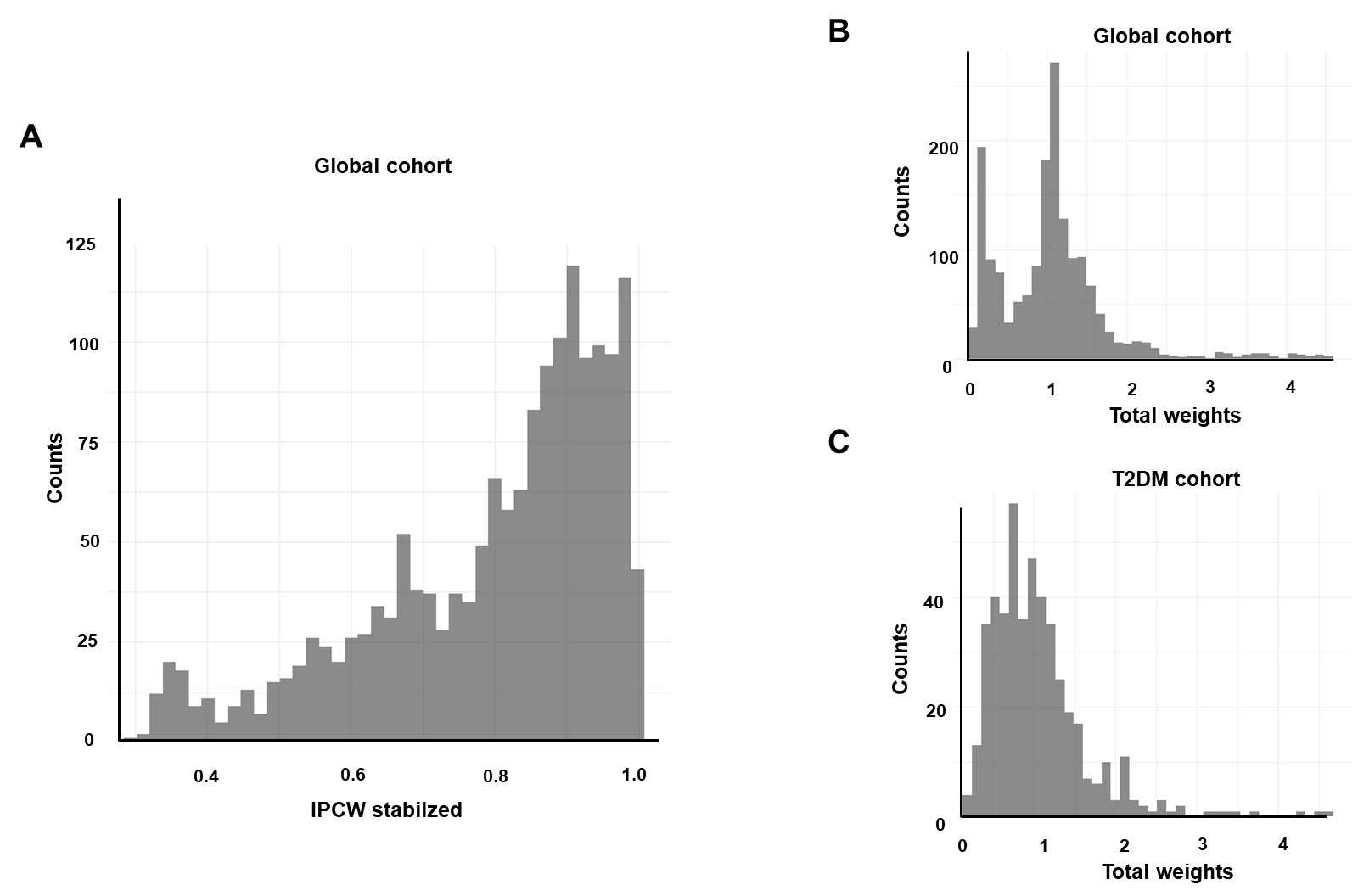
**

**Supplementary Figure S2. Distribution of inverse probability of censoring weights (IPCW) and combined analytic weights.** Panel (A) shows the distribution of stabilized IPCW derived from the overall censoring model. Panel (B) shows the distribution of total weights used in the overall weighted Cox model, derived as the product of overall stabilized inverse probability of treatment and censoring weights (IPTW × IPCW). Panel (C) shows the distribution of total weights used in the T2DM-restricted weighted Cox model, calculated as the product of T2DM-specific stabilized IPTW and the overall stabilized IPCW. Across panels, weight distributions were compact and well behaved, with no evidence of highly influential extreme values and only a limited right tail for the combined weights, supporting the stability of the weighting approach used in the weighted Cox proportional hazards analyses.

**
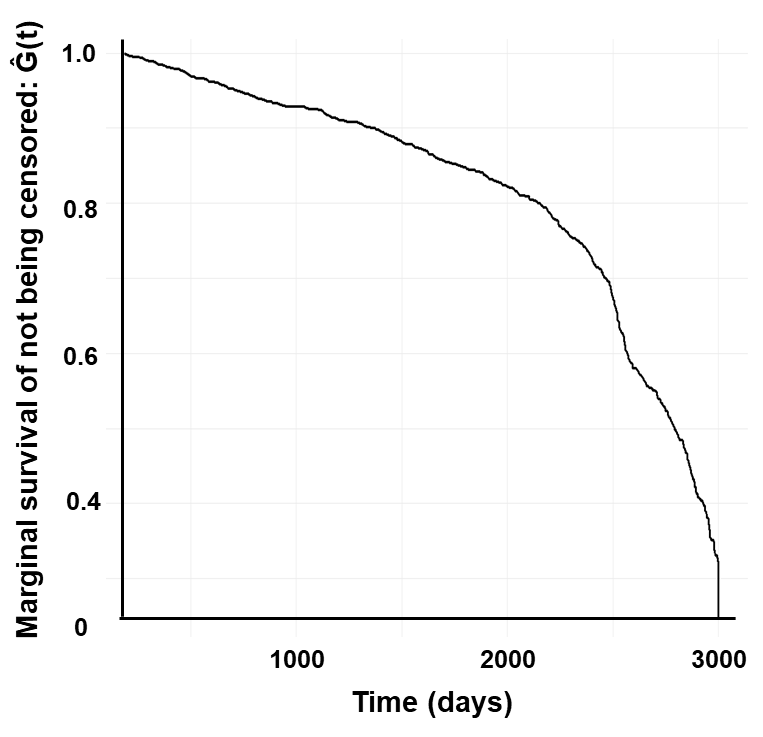
**

**Supplementary Figure S3. Marginal non-censoring survival function, G(t), estimated from the overall censoring model.** G(t) represents the probability of remaining uncensored over time, with censoring defined as loss to follow-up, non-HCC death, or non-HCC OLTbefore HCC occurrence. The resulting IPCW were used in both the overall and T2DM-restricted weighted Cox analyses. G(t) remained above 0.80 until approximately 2,500 days of follow-up.


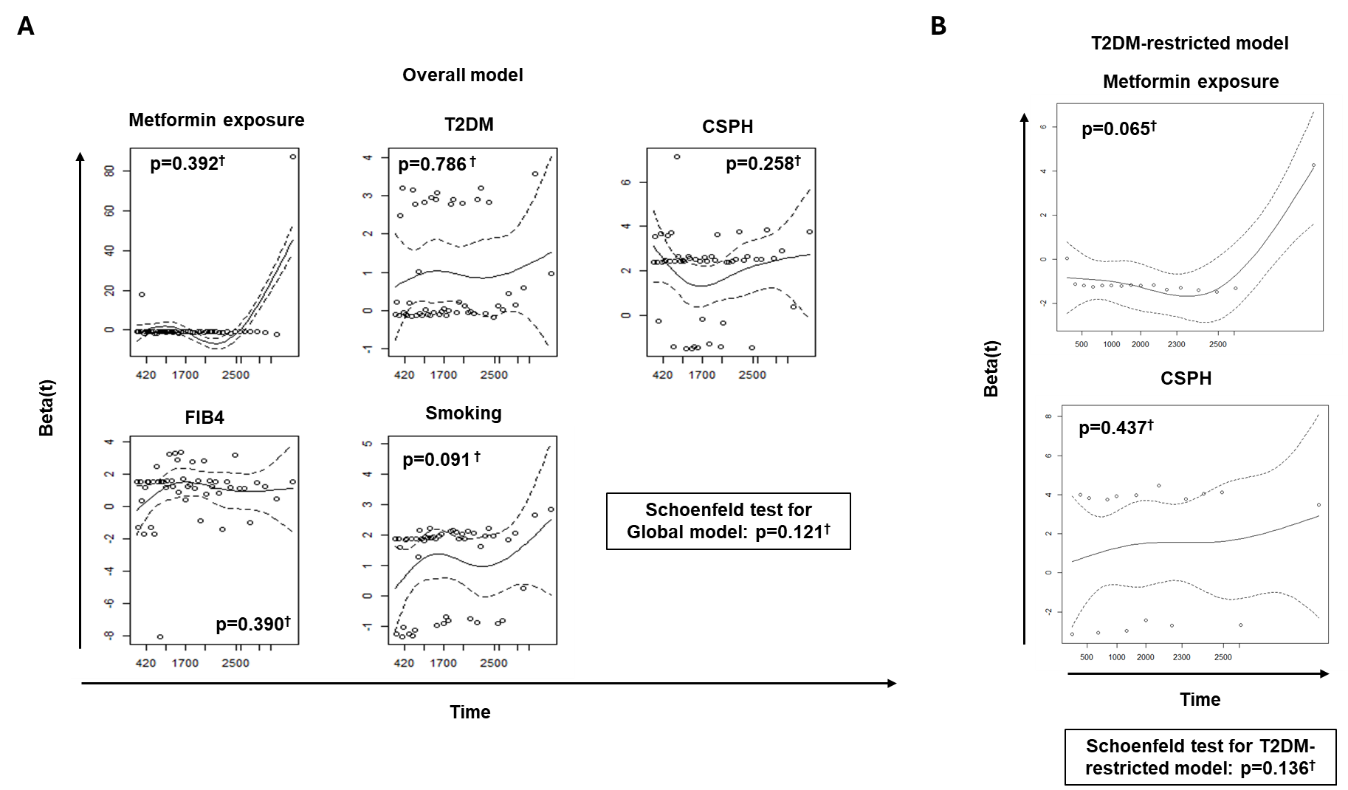


**Supplementary Figure S4. Schoenfeld residual diagnostics for the weighted multivariable Cox proportional hazards models.** Panel (A) shows the Schoenfeld residual plots and corresponding global and covariate-specific test results for the weighted overall model. Panel (B) presents the analogous diagnostics for the T2DM-restricted model. In both models, neither the global nor the covariate-specific Schoenfeld tests provided evidence of violation of the proportional hazards assumption, and visual inspection of the residual plots showed no clear sustained time-dependent trends. CSPH, clinically significant portal hypertension; T2DM, type 2 diabetes mellitus. ^†^Chi-square test.

**
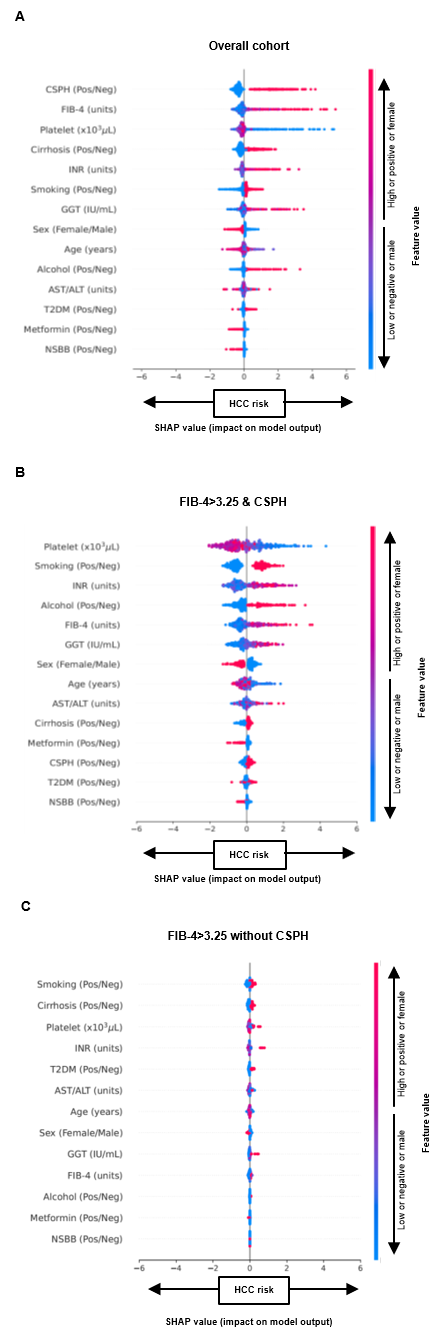
**

**Supplementary Figure S5. Random survival forest analysis of risk factors for hepatocellular carcinoma.** SHapley Additive exPlanations (SHAP) summary plots showing the relative importance and direction of baseline covariates in (A) the overall cohort, (B) the high-risk subgroup with FIB-4 >3.25 and clinically significan portal hypertension and (C) the subgroup with FIB-4 >3.25 without clinically significan portal hypertension. Each dot represents an individual patient, and the color denotes the value of the corresponding feature; for binary variables, red indicates higher values or presence of the characteristic, and blue indicates lower values or absence. For sex, red indicates female and blue indicates male. SHAP values on the x-axis indicate the magnitude and direction of each variable’s contribution to the predicted risk of hepatocellular carcinoma. ALT, alanine aminotransferase; AST, aspartate aminotransferase; CSPH, clinically significant portal hypertension; GGT, gamma-glutamyl transpeptidase; HCC, hepatocellular carcinoma; INR, international normalized ratio; NSBB, non-selective beta-blocker; T2DM, type 2 diabetes mellitus.

**
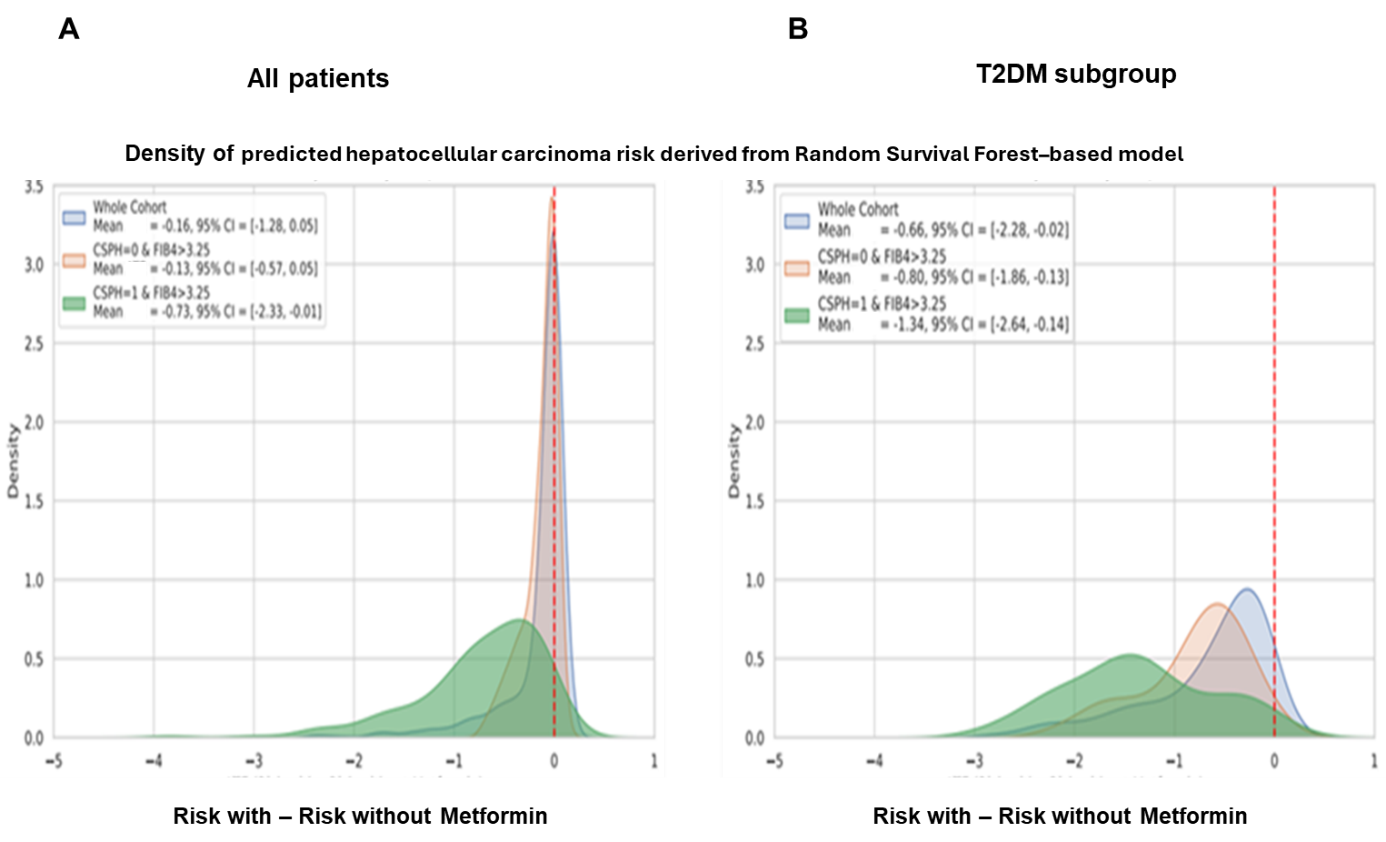
**

**Supplementary Figure S6. Estimated probability density functions of model-based average risk contrasts associated with metformin exposure.** Distributions represent differences in predicted hepatocellular carcinoma (HCC) risk derived from Random Survival Forest–based model predictions under hypothetical metformin exposure versus non-exposure scenarios. These contrasts are exploratory, population-specific, and model-dependent, and are intended to provide supportive insights rather than causal or confirmatory inference. Distributions are shown for the overall cohort (blue curve) and for subgroups defined by advanced fibrosis (FIB-4 >3.25) without clinically significant portal hypertension (CSPH) (red curve) and with CSPH (green curve) and, separately for (A) the entire study population and (B) patients with type 2 diabetes mellitus (T2DM). Negative values indicate lower model-predicted HCC risk associated with metformin exposure. CSPH=0, absence of clinically significant portal hypertension; CSPH=1, presence of clinically significant portal hypertension; T2DM, type 2 diabetes mellitus.

**Supplementary Tables**

**Supplementary Table S1. Concordance between the study-defined pragmatic CSPH variable and the Baveno VII liver stiffness-based rule-in criterion for clinically significant portal hypertension.**

| **Study-defined CSPH** | **Baveno VII LSM-based rule-in criterion for CSPH** | |  |
| --- | --- | --- | --- |
|  | **Absent:**  **LSM <25 kPa** | **Present:**  **LSM ≥25 kPa** | **Total** |
| **No** | 1,108 | 14 | **1,122** |
| **Yes** | 90 | 95 | **185** |
| **Total** | **1,198** | **109** | **1,307** |

Concordance was assessed among 1,307 patients with available baseline liver stiffness measurement. The Baveno VII liver stiffness-based rule-in criterion for CSPH was defined as liver stiffness measurement ≥25 kPa. Overall agreement between both classifications was 92.0%, with a Cohen’s κ of 0.61 (asymptotic standard error, 0.035; p<0.001), indicating moderate-to-substantial concordance. Among patients classified as having CSPH according to the study-defined pragmatic criteria, 90 of 185 patients (48.6%) did not fulfil the Baveno VII LSM ≥25 kPa rule-in threshold. This is consistent with the highly specific rule-in nature of this criterion, which is not intended as an exhaustive case-finding definition for all clinically relevant portal hypertension phenotypes. CSPH: clinically significant portal hypertension, LSM: liver stiffness measurement.

**Supplementary Table S2.** **Unweighted and weighted numbers of hepatocellular carcinoma events according to metformin exposure status in the weighted Cox models.**

**A: Overall cohort weighted Cox model**

| **Metformin exposure status** | **Number of patients** | **Unweighted HCC events (n)** | **Weighted HCC events*** |
| --- | --- | --- | --- |
| Never exposed to metformin | 1,310 | 48 | 84.4 |
| Ever exposed to metformin^†^ | 179 | 2 | 0.32 |

**B: T2DM-restricted weighted Cox model**

| **Metformin exposure status** | **Number of patients** | **Unweighted HCC events (n)** | **Weighted HCC events*** |
| --- | --- | --- | --- |
| Never exposed to metformin | 128 | 14 | 9.2 |
| Ever exposed to metformin^†^ | 179 | 2 | 1.68 |

*Weighted HCC events represent the sum of stabilized inverse probability of treatment and censoring weights across start–stop intervals in which hepatocellular carcinoma occurred. In Panel A, weights correspond to the overall cohort IPTW×IPCW; in Panel B, weights correspond to T2DM-specific IPTW×IPCW. Weighted counts do not correspond to additional observed cases and are shown for transparency regarding the contribution of each group to the weighted start–stop Cox models. ^†^“Ever exposed” indicates patients who contributed at least one interval with non-zero cumulative metformin exposure. In the weighted Cox models, metformin exposure was analysed as a time-updated cumulative variable rather than as a binary covariate.

**Supplementary Table S3.** **Distribution of antidiabetic treatments among T2DM according to metformin exposure status during follow-up.**

| **Antidiabetic treatment (N,%)** | **Non-metformin**  **(N=104)** | **Metformin**  **(N=204)** |
| --- | --- | --- |
| Only diet + exercise | 35 (33.6) | 0 (0) |
| Metformin alone | 0 (0) | 67 (32.8) |
| Metformin + Other OAD | 0 (0) | 85 (41.6) |
| Metformin + Insulin | 0 (0) | 19 (9.3) |
| Metformin + Other OAD + Insulin | 0 (0) | 33 (16.2) |
| Other OAD | 38 (36.5) | 0 |
| Other OAD + Insulin | 16 (15.4) | 0 |
| Insulin | 15 (14.4) | 0 |

Metformin exposure status was defined according to whether patients contributed metformin-exposed person-time during follow-up. OAD: oral anti-diabetic drug; T2DM: type 2 diabetes mellitus

**Supplementary Table S4.** **Sensitivity analyses replacing FIB-4 with liver stiffness measurement and clinical portal hypertension with the Baveno VII liver stiffness-based rule-in criterion for CSPH.**

| **Sensitivity analysis** | **Patients (n)** | **HCC events (n)** | **HR for cumulative metformin exposure** | **95% CI** | **p value** | **Global Schoenfeld p** |
| --- | --- | --- | --- | --- | --- | --- |
| FIB-4 replaced by LSM per 5 kPa | 1282 | 35 | 0.53 | 0.33–0.85 | 0.008 | 0.58 |
| CSPH study criteria replaced by Baveno VII CSPH rule-in criterion, LSM ≥25 kPa | 1282 | 35 | 0.52 | 0.31–0.89 | 0.017 | 0.68 |

Models were weighted using the prespecified analytic weights, stratified by sex, and fitted with robust variance estimation accounting for repeated start–stop intervals per patient. LSM, liver stiffness measurement; CSPH, clinically significant portal hypertension.

**Supplementary Table S5. Fine–Gray competing-risk regression models for hepatocellular carcinoma after sustained virologic response.**

**Panel A. Overall cohort Fine–Gray model**

| **Variable** | **Coefficient** | **sHR** | **95% CI** | **p value** |
| --- | --- | --- | --- | --- |
| **Metformin** | –2.12 | 0.12 | 0.04–0.37 | <0.001 |
| **CSPH** | 2.18 | 8.87 | 3.21–24.53 | <0.001 |
| **T2DM** | 1.09 | 2.98 | 1.42–6.23 | 0.004 |
| **FIB-4 1.45–3.25** | 1.21 | 3.36 | 1.07–10.53 | 0.037 |
| **FIB-4 >3.25** | 1.31 | 3.70 | 1.09–12.60 | 0.037 |
| **Active smoking** | 1.16 | 3.20 | 1.66–6.15 | <0.001 |

**Panel B. T2DM-restricted Fine–Gray model**

| **Variable** | **Coefficient** | **sHR** | **95% CI** | **p value** |
| --- | --- | --- | --- | --- |
| **Metformin** | –2.11 | 0.12 | 0.04–0.37 | <0.001 |
| **FIB-4 1.45–3.25** | 0.90 | 2.45 | 0.31–19.37 | 0.40 |
| **FIB-4 >3.25** | 0.49 | 1.64 | 0.14–19.48 | 0.70 |
| **CSPH** | 1.67 | 5.29 | 0.54–51.72 | 0.15 |
| **Active smoking** | 0.79 | 2.20 | 0.75–6.43 | 0.15 |

Subdistribution hazard ratios (sHRs) and 95% confidence intervals (CIs) were estimated using multivariable unweighted Fine–Gray competing-risk regression models for HCC, treating non-HCC death and non-HCC orthotopic liver transplantation before HCC occurrence as competing events. Metformin exposure was defined as post-SVR ever metformin use for this competing-risk sensitivity analysis. Panel A shows the overall cohort model, adjusted for metformin exposure, fibrosis severity, CSPH, T2DM, and active smoking. Panel B shows the prespecified T2DM-restricted model, adjusted for metformin exposure, fibrosis severity, CSPH, and active smoking. The overall model included 1,517 complete-case patients and was statistically significant (pseudo-likelihood ratio test, χ²=124, df=6; p<0.001). The T2DM-restricted model included 304 complete-case patients and showed lower precision because of the smaller number of HCC events (pseudo-likelihood ratio test, χ²=28.6, df=5). Because metformin exposure occurred almost exclusively among patients with T2DM, and the overall Fine–Gray model adjusted for T2DM status, the metformin subdistribution hazard ratio in the overall cohort reflects the within-T2DM contrast and was therefore very similar to that obtained in the T2DM-restricted model. CSPH, clinically significant portal hypertension; CI, confidence interval; HCC, hepatocellular carcinoma; OLT, orthotopic liver transplantation; sHR, subdistribution hazard ratio; SVR, sustained virologic response; T2DM, type 2 diabetes mellitus.

**References**

1. Younossi Z, Park H, Henry L, Adeyemi A, Stepanova M. Extrahepatic manifestations of hepatitis C: a meta-analysis of prevalence, quality of life, and economic burden. *Gastroenterology.* 2016;150:1599-1608.

2. Moreno-Juste A, Poblador-Plou B, Aza-Pascual-Salcedo M, et al. Initial therapy, regimen change, and persistence in a Spanish cohort of newly treated type 2 diabetes patients: a retrospective, observational study using real-world data. *Int J Environ Res Public Health.* 2020;17:3742.

3. European Association for the Study of the Liver. EASL clinical practice guidelines: management of hepatocellular carcinoma. *J Hepatol.* 2018;69:182-236.

4. Vittinghoff E, McCulloch CE. Relaxing the rule of ten events per variable in logistic and Cox regression. *Am J Epidemiol.* 2007;165:710-718.

5. Holland PW. Statistics and causal inference. *J Am Stat Assoc.* 1986;81:945-960.

6. Akiba T, Sano S, Yanase T, Ohta T, Koyama M. Optuna: a next-generation hyperparameter optimization framework. In: *Proceedings of the 25th ACM SIGKDD International Conference on Knowledge Discovery and Data Mining (KDD 2019).* New York, NY: Association for Computing Machinery; 2019. 2623–263*1*.
